# ProCogGraph: a graph-based mapping of cognate ligand domain interactions

**DOI:** 10.1093/bioadv/vbae161

**Published:** 2024-10-22

**Authors:** Matthew Crown, Matthew Bashton

**Affiliations:** Hub for Biotechnology in the Built Environment, Department of Applied Sciences, Faculty of Health and Life Sciences, Northumbria University, Newcastle upon Tyne NE1 8ST, United Kingdom; Hub for Biotechnology in the Built Environment, Department of Applied Sciences, Faculty of Health and Life Sciences, Northumbria University, Newcastle upon Tyne NE1 8ST, United Kingdom

## Abstract

**Motivation:**

Mappings of domain-cognate ligand interactions can enhance our understanding of the core concepts of evolution and be used to aid docking and protein design. Since the last available cognate-ligand domain database was released, the PDB has grown significantly and new tools are available for measuring similarity and determining contacts.

**Results:**

We present ProCogGraph, a graph database of cognate-ligand domain mappings in PDB structures. Building upon the work of the predecessor database, PROCOGNATE, we use data-driven approaches to develop thresholds and interaction modes. We explore new aspects of domain-cognate ligand interactions, including the chemical similarity of bound cognate ligands and how domain combinations influence cognate ligand binding. Finally, we use the graph to add specificity to partial EC IDs, showing that ProCogGraph can complete partial annotations systematically through assigned cognate ligands.

**Availability and implementation:**

The ProCogGraph pipeline, database and flat files are available at https://github.com/bashton-lab/ProCogGraph and https://doi.org/10.5281/zenodo.13165851.

## 1 Introduction

Protein domains are independently folding, stable, 3D protein substructures first proposed in the 1970s (Rao and Rossmann 1973). Various databases and methods exist for assigning domains in experimental and computationally predicted protein structures, including CATH, SCOP2, and Pfam (Andreeva *et al.* 2020, Mistry *et al.* 2021, Waman *et al.* 2024).

In biochemistry, a ligand is a molecule which reversibly binds to a protein, typically as part of the overall function of a protein. Ligands can be chemically and structurally diverse, including metal ions, small molecules, oligosaccharides, and other polypeptides. When considering enzymes, an endogenous ligand can be considered a naturally occurring ligand, e.g. ATP or glucose, but could also include naturally occurring inhibitors of enzymes; for instance, in Aspartate Transcarbamoylase cytidine triphosphate (CTP) acts as an inhibitor to the reaction in a feedback loop, by binding to an allosteric site and inducing a conformational change in the overall structure of the enzyme (Lipscomb and Kantrowitz 2012).

The term cognate ligand is used to describe the endogenous ligands a protein binds during normal function, and for enzymes describes the set of ligands (substrates, products, cofactors) involved in their normal function. In the case of experimentally determined structures, ligands are often present, which may be the expected in vivo cognate ligand, an exogenous analogue or inhibitor used to aid structural determination such as *N*-(phosphonoacetyl)-l-aspartate (PALA), a transition state analogue of the l-aspartate and carbamoyl phosphate substrates of the ATCase enzyme (Krause *et al.* 1987), or an artefact of crystallization, e.g. a buffer component such as glycerol.

Many different databases exist to document protein–ligand binding interfaces. The PDBe-Graph (Nair *et al.* 2021) is a graph database of residue level annotations for PDB structures, including protein–ligand contacts and also includes mappings to protein domains in CATH, SCOP, Pfam, and InterPro (Blum *et al.* 2021). Whilst the PDBe-graph contains a rich annotation, a limitation of this database is the lack of specific endpoints to extract protein domain specific interactions between a ligand and a protein. PDBsum is a web-server which provides summary information on PDB structures, including domain annotations and protein–ligand interactions, determined using HBPLUS, primarily focussed on schematics for visual analysis of proteins and provides a similarity measure between ligands in the structure and cognate substrates/products by comparing their maximum common substructure (MCS) (Laskowski *et al.* 2018). The iPfam database was a database of protein domain-domain and domain–ligand interactions based upon domain annotations from the Pfam domain database, and focussed on interactions directly observed in a structure, rather than mapping ligands to their cognate counterparts (Finn *et al.* 2014). BioLiP2 is a database of protein–ligand interactions specifically focused on identifying biologically relevant interactions through an artefact list of approximately 500 common artefact ligands (Zhang *et al.* 2024).

The PROCOGNATE database mapped domain-cognate ligand interactions to extract the biological relevance of domain–ligand interactions (Bashton *et al.* 2008). It included domain annotations from CATH, SCOP, and Pfam to provide both structural and sequence domain annotations, together with cognate ligand annotations from KEGG. These mappings have been used for evolutionary studies of domain and cofactor origins (Caetano-Anollés *et al.* 2012), to filter structures utilized in stability studies to only those containing cognate ligands (Juritz *et al.* 2012) and as a tool to curate collections of cognate ligands for other databases (Lopez *et al.* 2011). This database is no longer maintained, and in the meantime the PDB has continued to grow, adding to the number of structures without cognate ligand assignments.

Many of PROCOGNATE’s former features exist in other databases, e.g. PDBSum contains cognate ligand similarity and contacts between protein residues and the PDB ligand, and BioLiP2 annotates ligands and their receptor binding sites as well as filtering of nonbiologically relevant ligands through a curated list of additives. Whilst one or many of the original PROCOGNATE’s features have been replicated in subsequent databases, no complete cognate ligand–domain mapping has been available since the deprecation of PROCOGNATE after version 1.6—motivating the creation of our new database of cognate ligand mappings.

Representing the highly connected nature of protein domain–ligand interactions through a graph database may present the opportunity to draw new understandings from these interaction networks, e.g. understanding the central cognate ligands in biology and how different domains combine to interact with ligands. Graph databases are well suited to efficient searching of multi-level relationships, such as domain–ligand interactions at the level of domain families or superfamilies, or across EC classes. This graph modelling avoids the need to execute multiple joins across tables as in relational databases. In addition, by representing data in a graph, it is possible to quickly identify all domains which interact with a target cognate ligand, at the scale of the entire PDB. The hierarchical structure of graphs and their efficient path-searching capabilities also support dynamic interactions with the underlying data, enhancing usability, making graph databases particularly powerful for navigating and querying large-scale, complex biological datasets, such as the domain–ligand interactions being explored here.

To this end, we present a new analysis pipeline and graph database of cognate ligand–domain interactions, utilizing up-to-date methods and a more comprehensive range of compound and reaction databases to explore the ever-expanding set of structures available in the wwPDB and a flexible user interface to easily harness the knowledge of the underlying graph database.

## 2 Methods

### 2.1 The ProCogGraph pipeline

ProCogGraph integrates data from SIFTS (Velankar *et al.* 2013), which provides a mapping between the PDB and domain databases at residue level, the Chemical Component Dictionary (Westbrook *et al.* 2015), PDBe-modelserver (Varadi *et al.* 2022), which provides hydrogenated assemblies, and various reaction and cognate ligand structure databases. A NextFlow (Di Tommaso *et al.* 2017) pipeline is utilized to build the database from a target structure manifest and cognate ligand dataset (Supplementary Fig. S1). Enzyme structures are downloaded from relevant webservers (step 1a) using PDBe-KB preferred assemblies (accessed from https://ftp.ebi.ac.uk/pub/databases/pdbe-kb/complexes/assemblies_data.csv) and SIFTS EC annotation mapping (accessed from https://ftp.ebi.ac.uk/pub/databases/msd/sifts/csv/pdb_chain_enzyme.csv). Updated MMCIF files and SIFTS XML files are downloaded from the PDBe. Protonated assemblies are obtained from PDBe-modelserver, which enables hydrogen bonds to be determined within structures.

Cognate ligands are aggregated from KEGG, ChEBI, RHEA, GlyTouCan, and PubChem (Morgat *et al.* 2015, Hastings *et al.* 2016, Kanehisa *et al.* 2017, Kim *et al.* 2018, Fujita *et al.* 2021) (step 1b), using KEGG reaction IDs to map cognate ligands to EC reactions. SMILES strings are obtained for the various ligands, and these structures are loaded and processed using RDKit v2024.03.2 (Landrum *et al.* 2024). Cognate oligosaccharides are obtained through database cross-referencing between KEGG compound records and the GlyTouCan database GlycoCT sugar encoding. This is converted to a SMILES structure using the GlycanFormatCoverter and CSDB conversion APIs (Chernyshov and Toukach 2018, Tsuchiya *et al.* 2019). Cognate ligands are annotated with their cofactor status based on their ChEBI cross-references and their role, as assigned by ChEBI ontology. A benefit of the cognate ligand curation approach taken is that cognate ligands are collected for all EC numbers and not just those with a protein chain mapping, allowing for easy integration of new structures to the database in future releases (for discussion of the EC coverage and cognate ligand database overlap in ProCogGraph, see Supplementary Section S4). Bound entities (bound ligands and branched oligosaccharide entities) are identified in the structure from the updated mmCIF file. SMILES representations of bound ligands are obtained by cross-referencing ligand PDB codes to the Chemical Component Dictionary. A similar process to cognate oligosaccharide representations is employed for oligosaccharides in PDB structures. Where available, the WURCS representation of an oligosaccharide is obtained from the _pdbx_entity_branch_descriptor field and converted to SMILES as described above.

To identify a cognate ligand for a bound entity, a measure of similarity must be determined between the two structures. The Proportion of Atoms Residing in Identical Topology (PARITY) method is used to measure similarity between bound entities and cognate ligands (Tyzack *et al.* 2018), as it provides a fast, easily interpretable similarity based on common substructure between two ligands (1 = identical, 0 = no similarity). Various cutoffs for PARITY score have been used previously (Tyzack *et al.* 2018, Špačková *et al.* 2024), and in ProCogGraph, a minimum threshold is determined through analysis of random ligand pairs, to minimize the number of spurious matches. Five sets of 2000 randomly paired ligands were retrieved, and PARITY similarity calculated, with the 95th percentile score determined for each set. The mean 95th percentile score represents the typical similarity of noncognate ligand pairs (0.29 ± 0.01). To confidently assign a cognate ligand in ProCogGraph, the minimum required PARITY score is set 10% above this mean 95th percentile at 0.4 PARITY score. An important design decision in ProCogGraph is not to exclude multiple cognate ligands for the same bound entity. The minimum similarity threshold described above removes poor-quality matches; however, where multiple valid matches are available for a ligand, all results are presented to allow users to determine the appropriate “best” cognate ligand.

Contacts are determined using PDBe-arpeggio (Jubb *et al.* 2017) and mapped to protein domains from CATH, SCOP, Pfam, Superfamily, Gene3DSA, and SCOP2 (Wilson *et al.* 2009, Lees *et al.* 2012, Andreeva *et al.* 2020, Blum *et al.* 2021, Mistry *et al.* 2021, Waman *et al.* 2024) using SIFTS annotations (step 2, PROCESS_CONTACTS, see Supplementary Fig. S1). A minimum residue cutoff of three filters spurious contacts between residues and PDB ligands. This cutoff ensures that peripheral ligands to the assembly and sparse domain interactions with a ligand are removed, such as the DNA intercalating agent EVP, which binds to DNA in PDB 8J9V, and has a small number of contacts with the protein entities in the structure. Following the assignment of contacts, the domain interaction mode is assigned according to a set of cutoffs determined through evaluation of the distribution of contacts between domains and ligands. Interactions are based on the percentage of overall contacts identified to the ligand, regardless of type. This data is available within the graph as a property of the INTERACTS_WITH relationship. In addition, residue-level interactions between domains to a ligand are stored, providing a summary of the ligand binding interface of a domain.

Previously, PROCOGNATE used a binary interaction mode for protein domains and ligands, assigned as either “shared” or “non-shared” (Bashton *et al.* 2006). Analysis of the distribution of contacts from domains (Supplementary Fig. S2) shows that ligands are predominantly bound by a single domain, with 2- and 3-domain interactions present at a much lower level and 4+ domain interactions highly infrequent. One or two domain interactions make up >96% of all ligand interactions in ProCogGraph, across all domain database types (in the CATH database, single domain interactions make up 77% of all domain interactions). Gini index was used to investigate the inequality of interaction in multi-domain ligand interactions, which is commonly employed in financial settings to measure income inequality (Park and Kim 2021), and has been used in bioinformatics to describe gene expression level distribution (O’Hagan *et al.* 2018) and in chemical probe selection (Ursu *et al.* 2020), amongst others. A Gini value of ≥0.4 is commonly accepted to represent relative inequality, and the mean lowest domain contact percentage for interactions exhibiting relative inequality was 6.9% for 2-domain interactions, 7.5% for 3-domain interactions and 4.7% for 4-domain interactions in our analysis. On this basis, we set a “minor” domain interaction as any domain contributing fewer than 10% of contacts to a ligand. Minor interactions were verified by assessing the energetic (un)favourability of domain interaction using Surfaces (Teruel *et al*. 2023). Ten ligands containing “minor” interactions were randomly selected, together with ten ligands containing interactions between 10 and 20% of interactions, to represent the next closest group of domain contacts to the “minor” domains. An independent *t*-test was used to confirm that domains with 10%–20% (low partner) contacts have a significantly stronger interaction energy with ligands than minor domains (*t* = −5.05, *P* < 0.001; Supplementary Fig. S3). Where all except one domain interaction is classified as minor, this single domain is classified as either dominant (≥90%) or major (10%–90%). In ProCogGraph, we extend the “shared” definition of PROCOGNATE to increase the granularity of partner interactions. Two additional contact categories are defined to characterize these interactions—major partner (≥50% of all contacts within a ligand interaction) and partner (≥10% to <50% contacts). Supplementary Table S1 contains definitions for all domain interaction modes utilized in ProCogGraph, and their abundance in ProCogGraph relative to the total number of domain interactions for each domain database. Major partner and partner domains are the second most abundant interactions observed in ProCogGraph, behind exclusive interactions. Interactions of this type may, e.g. be cases of active site formation between domains or interactions where one domain is specialized to hold one moiety of a ligand and another holds a different moiety—see Section 3.4 for further exploration of combinatorial domain interactions.

ProCogGraph uses Neo4j v5.22.0 community edition as a database management system (DBMS), and NeoDash v2.4.8, a Neo4j plugin, is used for the dashboard interface. To ensure the interoperability of ProCogGraph regardless of the DBMS, ProCogGraph is distributed in flat file format alongside a build script for building the Neo4j version of the database, that can easily be adapted to other DBMS (Supplementary Fig. S4 gives an overview of the graph schema). The ProCogGraph mappings are also distributed in a flat-file format to allow for custom data exploration and integration with other tools and research. A domain-cognate ligand mapping file and domain-bound entity mapping file are provided for each domain database type. For integration of the cognate ligand similarity data into other tools and analyses, a PARITY similarity mapping file between PDB ligands and cognate ligands is also distributed. For details on running the ProCogGraph pipeline, database and dashboard, see Supplementary Section S1.

### 2.2 Analysis using ProCogGraph

Graph queries are performed using the Cypher language via the Neo4j Python driver and processed using Pandas v2.2.2. Where SMILES strings were returned from a query, these were processed using RDKit to generate Mol objects. Cognate ligand similarity space was explored using ligand MACCS 166 key fingerprints (Kuwahara and Gao 2021), generated via RDKit. These fingerprints were visualized using t-SNE (Maaten and Hinton 2008) to reduce dimensionality to 2D, with projections clustered using *k*-means clustering and the appropriate cluster number established through Silhouette analysis using scikit-learn v1.5.0 (Pedregosa *et al.* 2011). Similarity of cognate ligands was assessed using Tanimoto similarity of fingerprints at a threshold set based on analysis by Landrum using ligands from ChEMBL (Landrum 2021) demonstrating a Tanimoto threshold of ≥0.575 accounts for 90% of randomly paired molecules (strict threshold herein) and a threshold of ≥0.431 accounts for 70% of random pairs (permissive threshold herein). A promiscuous domain is defined in our analysis as a domain which has nonminor interactions with four or more cognate ligands, a threshold previously described, which avoids matching domains which interact with a cofactor, substrate and product from a single reaction (Bashton *et al.* 2006).

## 3 Results

**Figure 1.**
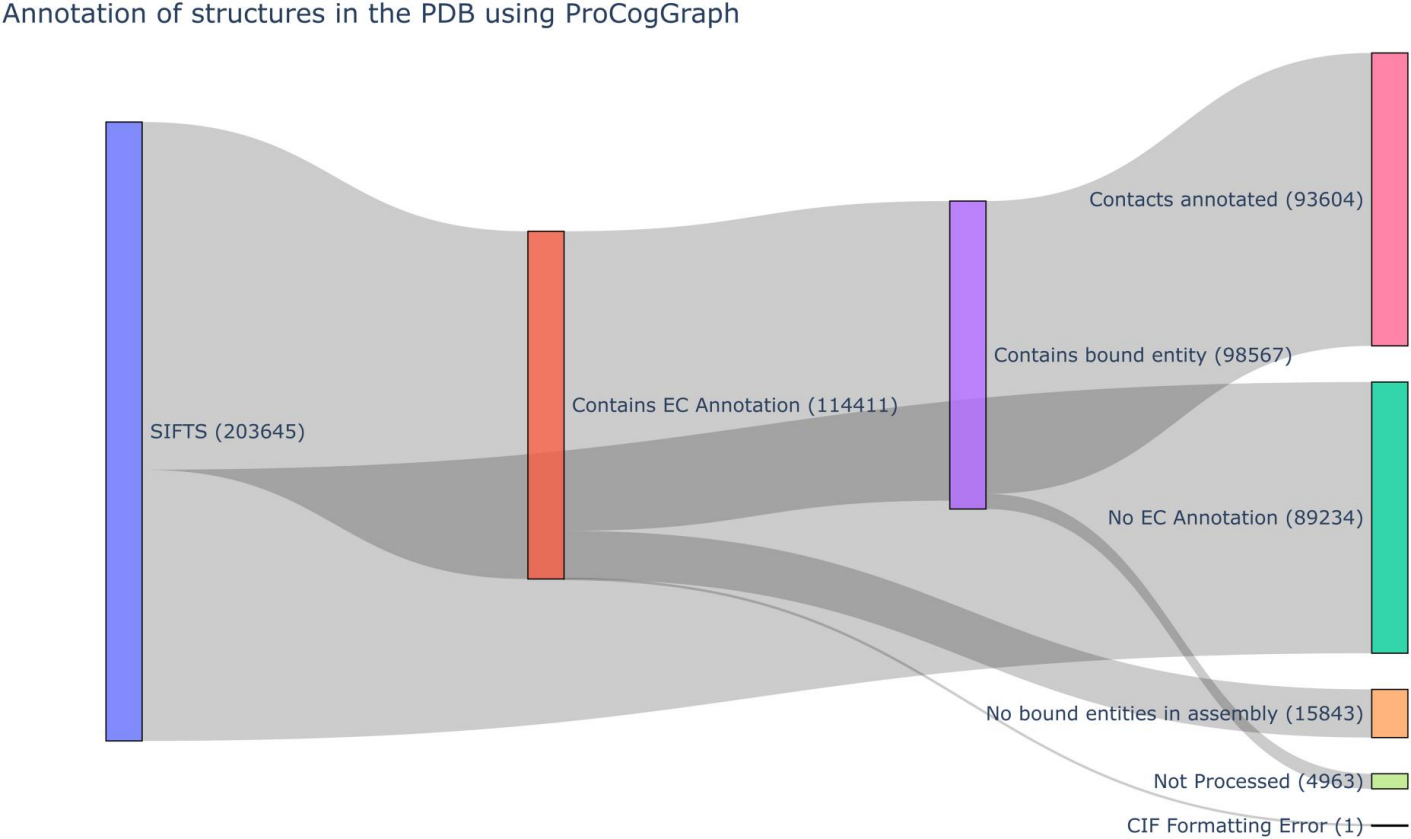
Procession of structures through the ProCogGraph pipeline. At each stage, some structures are lost due to either: no EC annotation, no bound entities or domains in structure, or structure contacts failing to meet criteria.

Figure 1 depicts the breakdown of structure annotation in ProCogGraph. Many structures are not enzymes/do not contain bound entities, and so are omitted. Overall, 95% of structures that can be annotated are included in the database. Supplementary Table S2 summarizes the domains, bound entities, and cognate ligand mappings in ProCogGraph compared to the last available version of PROCOGNATE (v1.6, March 2009). ProCogGraph significantly increases coverage, with four additional domain data sources added, an 11.2x increase in PDB structures, and a 4.5x increase in bound ligands with cognate ligand mapping.

### 3.1 The ProCogGraph dashboard

ProCogGraph is also accessible through a dashboard, which enables a rich interrogation of protein domain—cognate ligand mappings for all annotated structures and search functionalities by EC ID, cognate or bound entity, and protein domain. The dashboard allows users to filter matches by similarity score, access specific domain databases, e.g. CATH, SCOP2, Pfam, and filter interactions to, e.g. only view cognate ligand interactions with a domain which are exclusive. A detailed description of the ProCogGraph dashboard is available in Supplementary Section S2 and Supplementary Fig. S5.

### 3.2 Cognate ligands in ProCogGraph

At the minimum similarity threshold, 169 693/489 661 bound entities (representing 7408 structurally unique bound entity descriptors) have at least one mapping to cognate ligands, of which 73 590 are perfect matches. In line with previously reported cognate ligand mappings (Bashton *et al.* 2006), >50% (41 945) of these matches are to cofactors. To understand the chemical space cognate ligands occupy, assigned cognate ligands were clustered using their chemical fingerprint by *k*-means clustering (*n* = 14, mean silhouette coefficient 0.51—see Supplementary Fig. S6). Some well-distinguished clusters are apparent within the projection, including clusters 4, 5, and 11–13. Inspection of the ligands within these clusters shows that they correspond to: nucleotides/derivatives (cluster 11), sugars (cluster 5), phosphorylated sugars and metabolites (cluster 4), pyrroles (cluster 12), and Coenzyme A/conjugates (cluster 13). In addition, cluster 9 (silhouette score 0.45) contains many amino acids and their intermediates, such as l-saccharopine, a compound produced in the degradation of lysine.

**Figure 2.**
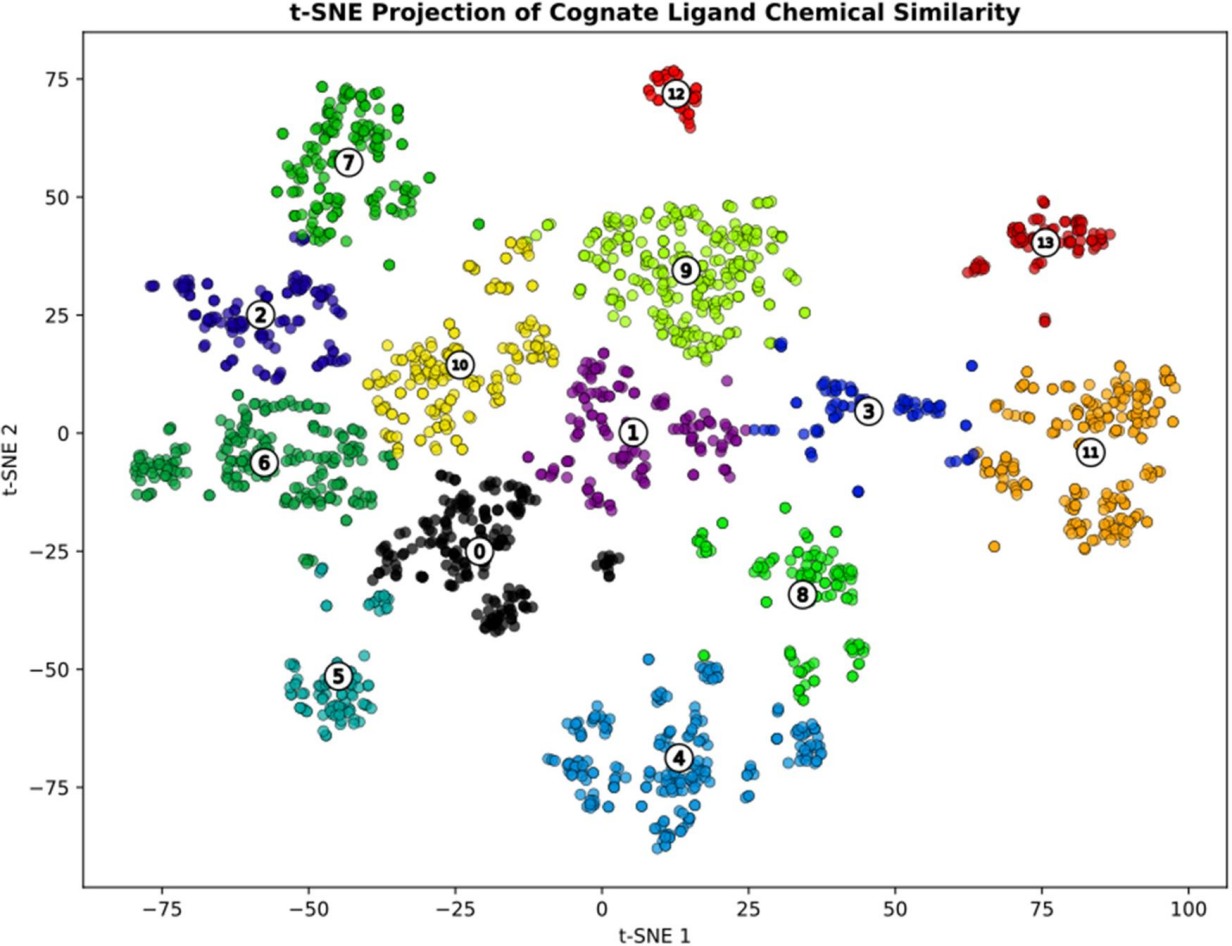
Cognate ligand cluster visualization using t-SNE. Each point represents a unique cognate ligand, which is coloured according to its assigned cluster using KMeans clustering. Cluster centroids are annotated with the cluster number. Not all clusters within the ordination have clearly similar structure or function—however it can be seen that cluster 4 corresponds to phosphorylated sugars and metabolites, cluster 5 to sugars, cluster 9 to amino acids/intermediates, cluster 11 to nucleotides/derivatives, cluster 12 to pyrroles and cluster 13 to Coenzyme A/conjugates. The remaining clusters are defined as the cognate substrate/product space.

The cognate ligand chemical space (Fig. 2) reflects the diversity seen in assigned perfect matches: many cognate ligands cluster closely together and seem to cluster based on their core cofactor structures, e.g. pyrroles (cluster 12) or Coenzyme A (cluster 13), with the remaining clusters less well defined and representing the overall product/substrate space of cognate ligand binding. The clustering of ligands is used in subsequent sections to relate chemical similarity of cognate ligands to domain binding.

Only 25% of the total number of bound entity descriptors (7408/29 635) have a mapping to a cognate ligand. Ligands that are never mapped may be genuine noncognate ligands, map to a cognate ligand not present in any of the cognate ligand databases, or not meet the minimum similarity threshold. Of the 22 086 bound entity descriptors with no cognate ligand match, the top 10 ligands account for 8% of all bound entity occurrences of these descriptors, and include common membrane components and ions used as part of substrate analogues (Supplementary Section S3 and Supplementary Table S3).

### 3.3 Chemical diversity in domain–ligand interactions

Within ProCogGraph, 460 promiscuous superfamilies are identified. One aspect of cognate ligand interaction left unexplored by simply considering the promiscuity of a domain interaction is the chemical (dis)similarity between the cognate ligand set. To address this, the within and between group chemical diversity of cognate ligands bound by superfamilies was explored using cognate ligand fingerprints. In addition to promiscuous superfamilies, superfamilies can be explored as either “specialized”—where the superfamily interacts with highly similar ligands only, or “generalized”—where the cognate ligands a superfamily interacts with show a high degree of dissimilarity. Of the 460 promiscuous superfamilies, only 15.7% interact with chemically similar (Tanimoto ≥0.575) cognate ligands within ProCogGraph, meaning that the majority of promiscuous domains are also “generalized” domains. In promiscuous superfamilies, the most “generalized” superfamilies tend to bind across the substrate ligand space (Supplementary Fig. S7). A promiscuous superfamily may be “specialized” because of a very specialized function, or lack of cognate ligand or bound entity representation in the underlying dataset. Supplementary Figure S8 shows the cognate ligands bound by the five most “specialized” promiscuous superfamilies which display the lowest intra-superfamily chemical dissimilarity—these “specialized” domains interact with distinct clusters within the ligand chemical space, particularly those clusters identified as cofactors, e.g. cluster 11/12. The difference between specialized and generalized superfamilies is also reflected in the diversity of EC numbers that domains from a superfamily are annotated as belonging to. Considering the ten most specialized and ten most generalized superfamilies, it can be seen that the specialized superfamilies have a much lower diversity of EC numbers compared to generalized superfamilies (mean 3.8 EC numbers compared to mean 324.6 EC numbers) suggesting that generalized superfamilies are highly multifunctional, rather than performing the same function on diverse ligands (see Supplementary Table S4).

**Figure 3.**
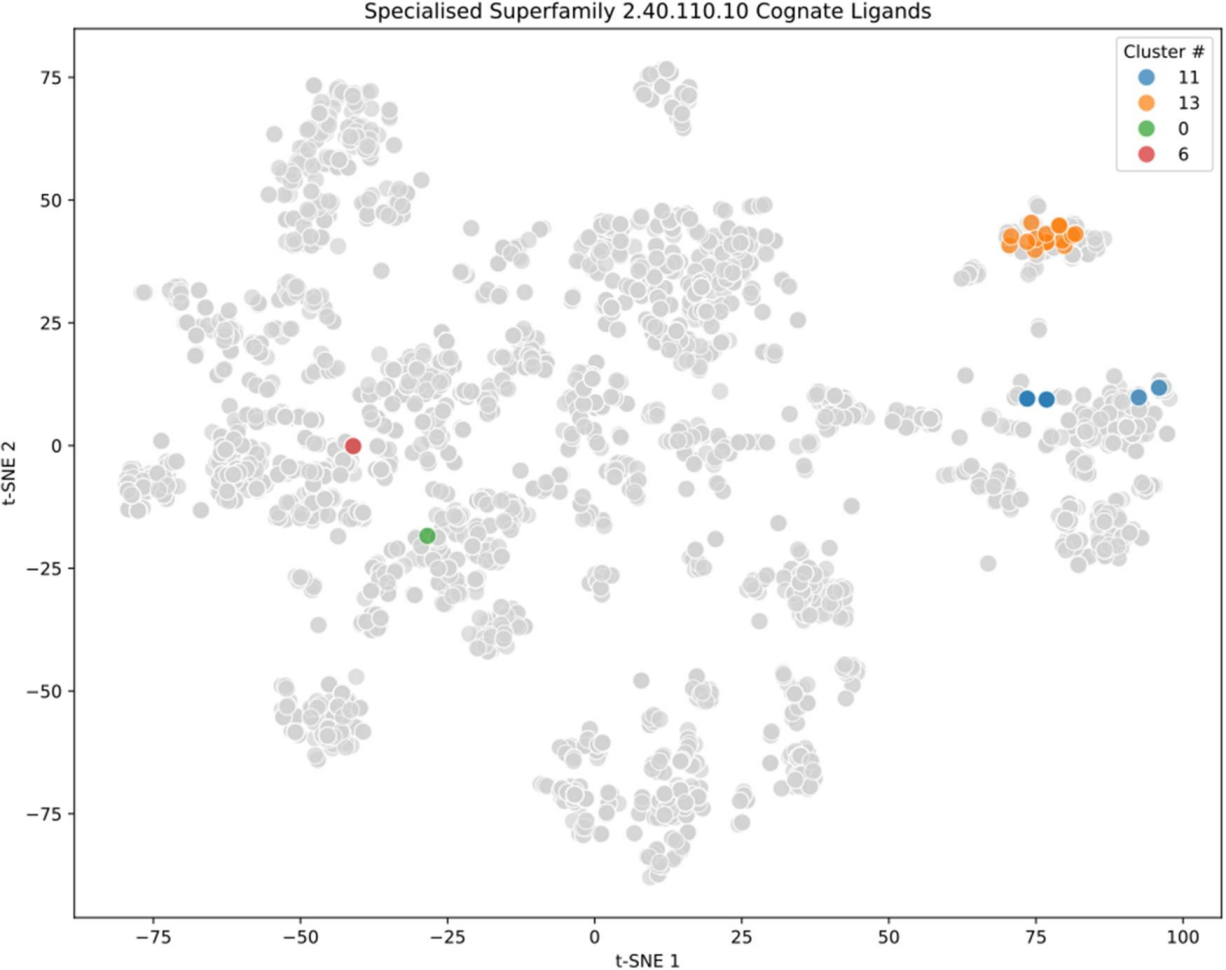
Specialized cognate ligand interactions. t-SNE visualization of cognate ligands from specialized SF 2.40.110.10 (Butyryl-CoA Dehydrogenase, subunit A, domain 2), coloured, compared to all unique ProCogGraph cognate ligands (grey). Ligands are clustered based on their chemical similarity and coloured according to cluster number. Cluster 13 (orange) consists primarily of CoA/derivative ligands, while Cluster 11 (blue) contains FAD/NAD/derivative ligands with remaining cognate ligands in clusters 0 (green) and 6 (red). The high Tanimoto score (0.75 ± 0.30) indicates a high similarity among the ligands within each cluster.

As previously discussed, a promiscuous superfamily is generally also a “generalized” superfamily. Supplementary Table S5 shows the most promiscuous superfamilies within ProCogGraph with good agreement compared to the previously reported promiscuous domains defined by PROCOGNATE (Bashton *et al.* 2006, Table 1). However, examples of promiscuous superfamilies with specialized ligand binding interaction can also be seen. SF 2.40.110.10 (Butyryl-CoA Dehydrogenase, subunit A, domain 2) interacts with 23 potential cognate ligands which have a high (0.75 ± 0.30 Tanimoto score) similarity (Fig. 3 below), the majority of which are CoA/derivative ligands (cluster 13), with a small subset of FAD/NAD/derivative ligands (cluster 11).

### 3.4 Combinatorial domain interactions in ProCogGraph

Domain contexts have been previously explored manually in a select set of circumstances (Bashton *et al.* 2006, Bashton and Chothia 2007), and have been shown to influence the interaction of domains with different cognate ligands. This was investigated by identifying the cognate ligands bound in a “non-shared” manner by a domain and the collection of other domains present in the protein. In addition to these interactions, in which a single domain is involved in ligand binding, multiple domains may interact with a single ligand. To explore this type of interaction, a combinatorial domain interaction is defined here as the collection of domains (more than one domain), at homologous superfamily level for CATH domains, which interact with a given PDB ligand in a nonminor manner, and for which the PDB ligand has a cognate ligand mapping with similarity score ≥0.4.

A total of 1003 unique domain combinations are observed when using CATH domain data. There are various potential ways in which a combinatorial domain interaction may impact ligand interaction—it may be transient/have no impact on the observed cognate ligands bound in the interaction compared to the individual domains—potentially functioning to bring together a substrate and cofactor or two substrates; it may enable a different or expanded or more specific range of interactions to occur; or improve the interaction of a protein to a ligand also commonly interacted with by one of the partner domains in an exclusive manner.

**Figure 4.**
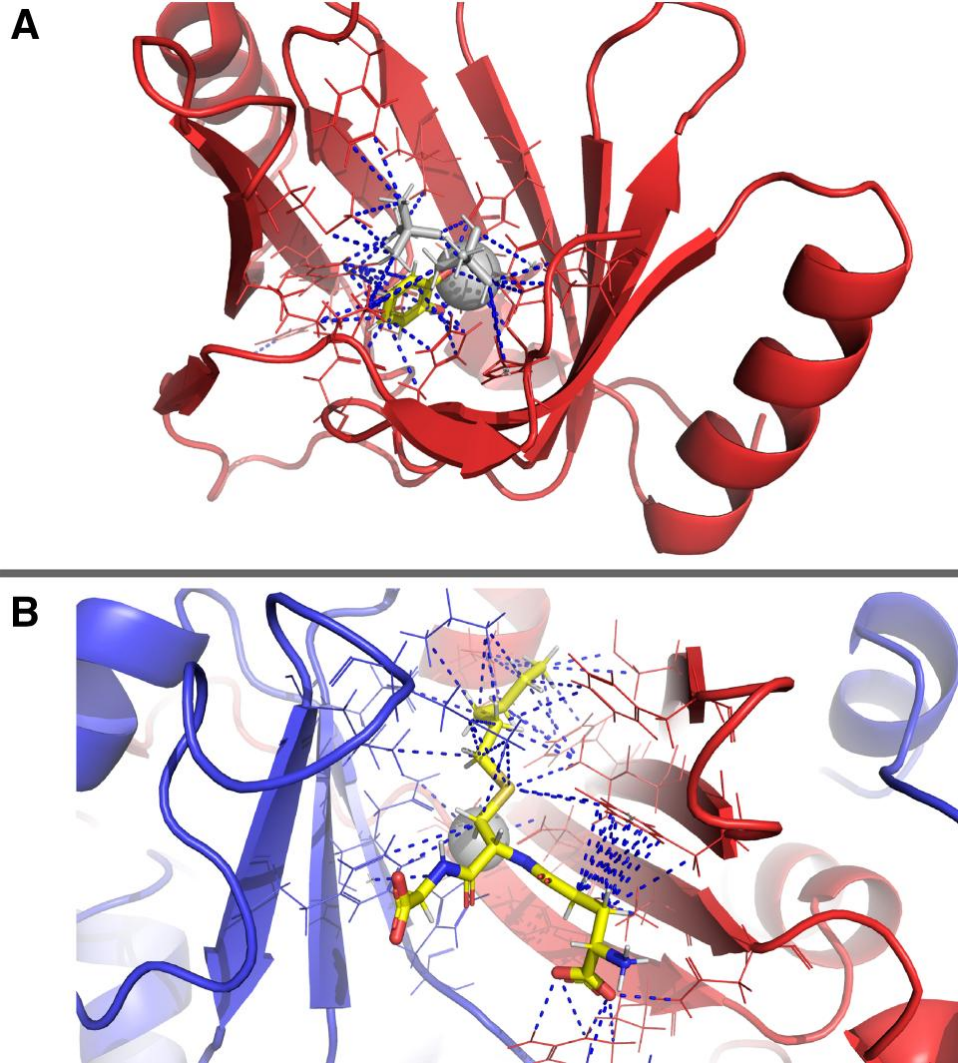
Exclusive and partner interactions of domain 2,3-Dihydroxybiphenyl 1,2-Dioxygenase, domain 1 (CATH Code 3.10.180.10). Blue dashed lines indicate contacts identified by PDBe-arpeggio. (A) Exclusive interaction with catechol (stick representation with carbon atoms coloured yellow, cognate = 3-chlorocatechol, 0.89 PARITY score) within the beta-barrel of the domain (PDB 1KND). Zinc ion and tertiary-butyl alcohol ligands are coloured grey. (B) Partner interaction of two instances of the domain (PDB 1BH5), coming together to form the enzyme active site for PDB ligand s-hexylglutathione (stick representation with carbon atoms coloured yellow, cognate = (R)-S-Lactoylglutathione, 0.82 PARITY score). Zinc ion highlighted in grey as a space filling representation.

A duplicate major partner-partner interaction is observed with the superfamily “2,3-Dihydroxybiphenyl 1,2-Dioxygenase, domain 1” (CATH 3.10.180.10). In some structures, this domain interacts exclusively with its bound ligand through a beta-barrel structure from a single domain (Serre *et al.* 1999)—see Fig. 4A. The duplicate partner interaction observed between two of these domains results in a structure where ligand binding is at the dimer interface, and the two domains each provide beta sheets to the beta barrel-like coordination of the ligand (Fig. 4B).

Here, the shared modality of binding enables a larger and more complicated binding pocket at the domain dimer interface, which is able to bind to the larger and more chemically complex glutathione, a tripeptide, through the interface of beta sheets from two domains versus the smaller aromatic diol which is bound in the core of a barrel-like structure in exclusive interactions of the domain superfamily. Supplementary Figure S9 shows the topological secondary structure organization of domains in both 1KND and 1BH5. A possible evolutionary mechanism for this difference is a domain duplication from an original single domain (like that in 1KND) and subsequent divergence modifying the two domains so that they form a functional dimer interface when paired, such as that seen in 1BH5.

Exclusive interactions are observed to aromatic diol cognate ligands such as Naphthalene-1,2-diol and Biphenyl-2,3-diol—to be expected due to the EC annotation of these structures as EC 1.13.11.-(involved in the cleavage of aromatic compounds). Conversely, partner interactions are observed to glutathione and glutamate, in structures annotated as EC 4.4.1.-(carbon-sulfur lyases). Here, glutathione, a tripeptide, is a more complex molecule than the simple aromatic structure bound exclusively and includes multiple functional groups, which may necessitate the larger binding pocket afforded by a multi-domain interaction mode.

A partner interaction between superfamily “Butyryl-CoA Dehydrogenase, subunit A, domain 3” (1.20.140.10) and superfamily “Butyryl-CoA Dehydrogenase, subunit A, domain 2” (2.40.110.10) demonstrates how two domains can act in combination to bind a new cognate ligand. In isolation, these domains interact with ligands such as propanoyl-CoA in a prolyl oxidase structure (PDB 6CY8, EC 1.3.8.1), and 4-hydroxyphenylacetate substrate in 4-Hydroxyphenylacetate 3-hydroxylase (EC 1.14.14.9) structures (CATH 1.20.140.10 PDB 2JBT, CATH 2.40.110.10 PDB 2YYM). In combination, these domains interact with coenzyme FAD, creating a binding pocket, which is used to activate O_2_ for the incorporation of oxygen into the substrate (Paul *et al*. 2021). This demonstrates how combinatorial domain interactions can be essential to enzyme function. All three domains are involved in the interaction, but bind distinct components of the FAD molecule. Supplementary Figure S10 shows two examples of this coenzyme binding pocket, in a p-hydroxyphenylacetate hydroxylase (Supplementary Fig. S10A, PDB 2JBS) and in a butryl-CoA dehydrogenase (Supplementary Fig. S10B, PDB 1BUC). In PDB 1BUC, the adenine moiety of the FAD molecule interacts with residues from superfamily 1.20.140.10 domains in two chains. In contrast, most interactions with the isoalloxazine ring are from a single superfamily 2.40.110.10 domain. Thus, in this instance, the different components of the domain combination each bind a different chemical moiety of FAD.

### 3.5 EC ID completion via cognate ligand detection

**Figure 5.**
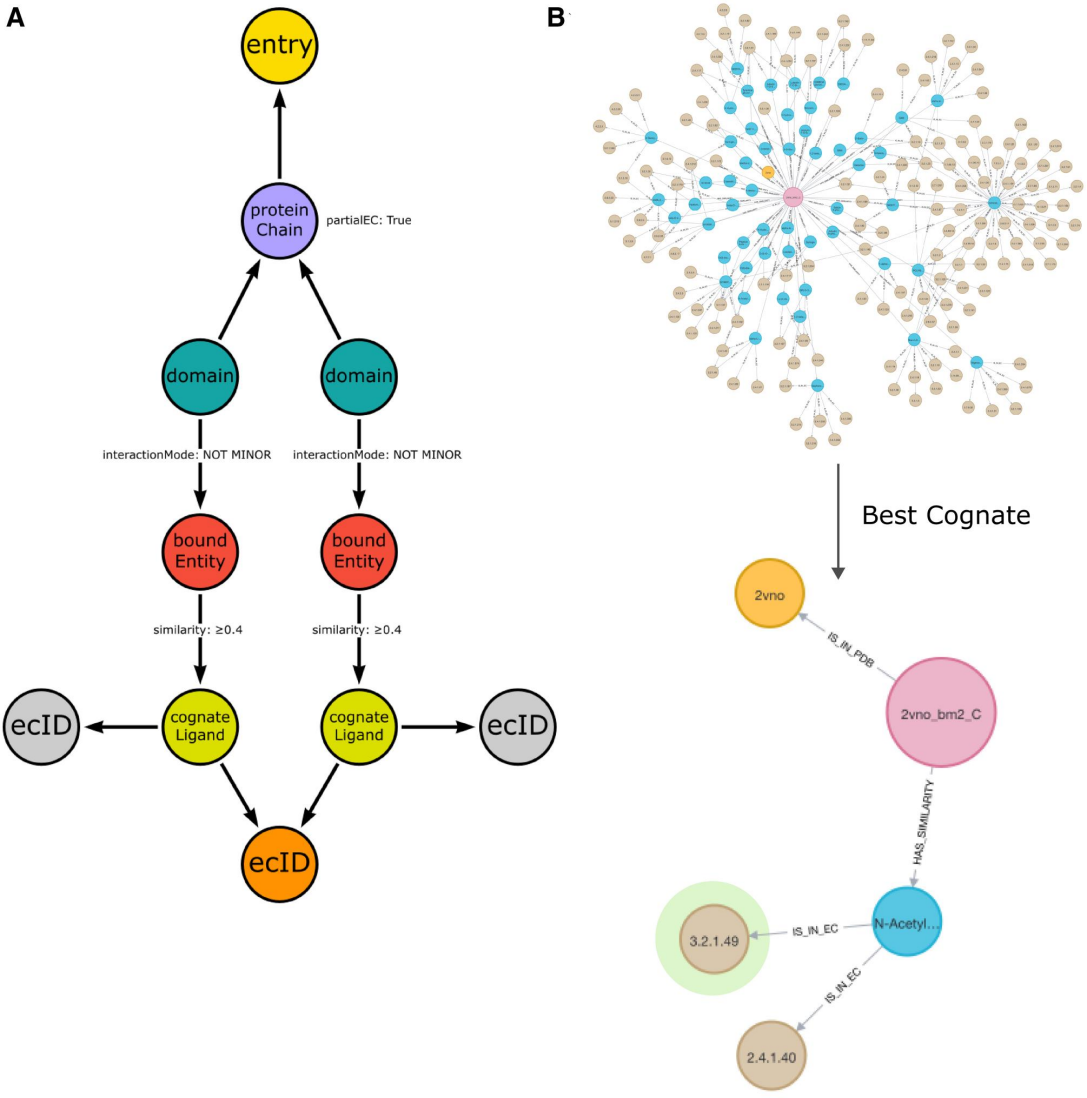
Cognate ligand mapping to determine exact EC IDs. (A) The graph schema for matching EC IDs with cognate ligands. For protein chains with partial ECs, nonminor domain interactions to bound entities are traced to cognate ligands with similarity above 0.4. An annotation is made if all cognate ligands share the same EC when multiple bound entities are present. (B) For PDB 2VNO, from partial EC 3.2.1, 54 cognate ligands are matched. The highest similarity ligand corresponds to EC 3.2.1.49.

ProCogGraph expands cognate ligand comparisons to include all EC IDs for a partial EC annotation, e.g. 2.1.1.-(methyltransferases). Using the likely cognate ligand bound by a domain, a specific EC number can be assigned to these structures. 24 041 protein chains across 15 587 PDB structures contain a partial EC annotation, of which 7923 chains (5545 PDB structures) have a domain ligand interaction mapping that can be traced to a cognate ligand using our methodology (Fig. 5A).

With these mappings, specific EC IDs can be assigned to protein chains by exploring the similarity of all cognate ligands in the undefined EC range to those bound in the PDB structure, improving the annotation of structures in the PDB and allowing greater insight into the functional roles of structures. In ProCogGraph, 1916 protein chains can be mapped through cognate ligands to specific EC numbers. Occasionally, SIFTS assigns a specific and partial EC number to a protein chain—604 protein chains with partial EC annotation also contain a specific EC, and so are excluded. This leaves 1312 protein chains in which the specific enzymatic function of the chain can be assigned through cognate ligand and domain interaction matching. 23.9% (314) of these chains were mapped to the specific EC ID through perfect bound entity-cognate ligand matches, i.e. the bound ligand was the cognate ligand.

As an example of the potential of this annotation method, in the PDB structure 2VNO, the assigned EC number via SIFTS was 3.2.1., corresponding to Glycosidases, of which there are over 100 EC IDs. The similarity of the bound ligands to cognate ligands from all potential EC IDs is compared, matching 54 cognate ligands across 150 EC IDs. For each bound entity, a “best cognate” property is assigned to the cognate ligand(s) which have the highest similarity score, only 1 cognate ligand has the highest similarity, resulting in an assignment of EC 3.2.1.49, Cleavage of nonreducing alpha-(1->3)-N-acetylgalactosamine residues from human blood group A and AB mucin glycoproteins (Fig. 5B).

Often, these EC matches are evident in publications, e.g. in PDB 2VNO the specificity of the structure for blood group antigens is discussed. However, using a cognate ligand assignment approach allows systematic annotation of EC IDs. Whilst assigned EC IDs should always be manually verified to ensure accurate mapping, such a method enables large-scale completion of EC identifiers where this cognate ligand mapping is available. This approach works well to assign granular EC IDs to ligands based on observed mappings to cognate ligand substrates/products of reactions and complements other tools for annotation, such as the structure-only based DeepFRI (Gligorijević *et al*. 2021), enabling the highest-level EC ID annotation where structure alone cannot differentiate between fourth level substrate specific identifiers.

## 4 Discussion

Here, we describe a new cognate ligand–domain interaction database, ProCogGraph, which builds upon its ancestor, PROCOGNATE, by extending coverage 11-fold across an expanded set of databases, including cognate ligands from several different databases, incorporating matches to sugars not previously covered, and employing a new data-driven methodology for defining domain–ligand interaction modes and cognate similarity thresholds. Alongside this, a highly customizable dashboard interface is provided, through which users can tailor data to suit their needs, searching for specific structures, EC numbers, cognate ligands or PDB ligands, together with a number of filters for similarity strictness and interaction modes.

We highlight new approaches to considering cognate ligand mapping, exploring the chemical space occupied by cognate ligands, and introducing the idea of specialized domains binding a chemically narrow range of ligands and generalized domains binding a chemically broad range of ligands. We show many of the specialized domains are involved in binding to cofactors, whereas generalized domains are more frequently involved in binding to the substrate/product chemical space. In addition, the combinatorial interaction of domains with ligands and the impact this has on the type of ligand bound is explored. Different mechanisms for combinatorial interaction are highlighted—combinatorial interactions may create binding pockets for new ligands not otherwise seen with exclusive domain interactions or domains may be duplicated, resulting in increased flexibility and altered ligand binding. The knowledge highlighted here could be of use in protein engineering to enable design of proteins able to bind to target ligands flexibly.

We explore the potential reasons for bound entities not matching to cognate ligands and find that many of the most frequently unmatched ligands are common membrane components or compounds used alongside biologically relevant ligands during crystallization, e.g. beryllium trifluoride with ADP. The most commonly unmatched ligand is Chlorophyll A due to its lack of presence as a cofactor in reaction schemes.

Overall, the cognate ligand–domain mapping ProCogGraph provides is important for continuing to evaluate the core ideas behind cognate ligand domain interactions. It also provides a new interface, metrics, and concepts such as domain generalization and specialization, based upon the chemical similarity of cognate ligands, through which to evaluate domains.

## Supplementary Material

vbae161_Supplementary_Data

## Data Availability

ProCogGraph is freely available and released under an MIT licence. ProCogGraph source code is available at https://github.com/bashton-lab/ProCogGraph and is deposited in Zenodo (https://doi.org/10.5281/zenodo.13249841). Data is archived in Zenodo (https://zenodo.org/doi/10.5281/zenodo.13165851).
