# Supplementary material for: ProCogGraph: a graph-based mapping of cognate ligand domain interactions
Source: Bioinform Adv. 2024 Oct 22;4(1):vbae161. doi: 10.1093/bioadv/vbae161 (PMC11561043; doi:10.1093/bioadv/vbae161)
Supplement: vbae161_Supplementary_Data [file vbae161_supplementary_data.zip › RESUB_ProCogGraph Paper Supplementary Text.docx]

## Supplementary Text

### S1. ProCogGraph Installation and Setup.

**Installation and Running the Database**

As part of the installation process, the latest flat files are downloaded from Zenodo. These files are currently 175.9 MB, and so the download may take some time depending on your internet connection. The total size of the database once built is approximately 4GB - ensure you have sufficient disk space available before beginning the install.

NOTE: Currently, the NeoDash Docker image does not contain a build for arm based Mac devices. There is an open issue (https://github.com/neo4j-labs/neodash/issues/754) in NeoDash related to this, and until it is fixed by the developers, ProCogGraph cannot be setup via Docker on arm-based Mac devices. Therefore execution of Docker steps is limited to x86 Mac devices. ProCogGraph can still be installed directly on arm-based Mac devices by following the steps in the installation guide and using the web-hosted (by Neo4j) NeoDash web app.

*Demo Mode*

To facilitate understanding of the types of data available in ProCogGraph, a demo database is available to access online. This database can be accessed from the NeoDash web app (http://neodash.graphapp.io/). You will need to download the dashboard_remote.json file from the procogdash directory, or copy its contents, in order to use this mode. To access the database, navigate to the NeoDash site (http://neodash.graphapp.io/) and use the following parameters on the splash screen:

- Protocol: neo4j+s

- Hostname: neo4j+s://4135bfe1.databases.neo4j.io

- Port: 7687

- Username: neo4j

- Password: hSIIDVT4T8KDFMHYuDSoxKovBSiKk5xlSSZhv8n4TfQ

Once connected, you can import the dashboard by clicking the expand menu icon in the bottom left of the screen, and selecting the `+` icon in the top right of this menu. Here, you have the option to import a dashboard, either by pasting the code from the dashboard_remote.json file or uploading it as a file. This will load the dashboard into the NeoDash web app, allowing you to explore the database.

A video walkthrough of this process, and the features of the dashboard, is available at <https://www.youtube.com/watch?v=qObmQNvKPnw>.

*Docker*

ProCogGraph is both a pipeline for analysis of structures and a database of cognate ligand-domain mappings. To get started, the easiest method, described below, is to run ProCogGraph in a Docker container - for installation instructions for the database on bare metal, and for running the Nextflow pipeline see the [installation](docs/installation.md) guide.

1. Download and install Docker from the Docker website (<https://www.docker.com/get-started>)
2. Clone the ProCogGraph repository:
    git clone m-crown/ProCogGraph
    cd ProCogGraph
3. Run the setup script to download the latest flat files and create the necessary directories and Docker compose files if running on Linux/OSX:
    ./setup_docker_linux.sh

   or for Windows (in Powershell with administrative access)
    Set-ExecutionPolicy Unrestricted
    ./setup_docker_windows.ps1

   This script creates the necessary directories for setting up the database, downloads the latest flat files from Zenodo and produces two yaml files on Linux/MACOS, and two powershell files on windows, one to build the database (run first time only) and one to run the database (run each time you want to start the database).
4. Run the build command:
   Linux/MACOS:
    docker compose -f compose-build.yml up
   Windows:
    ./run_build.ps1
5. Run the database:
   Linux/MACOS:
    docker compose -f compose-run.yml up
   Windows:
    Set-ExecutionPolicy Unrestricted
    ./run_services.ps1

   After running the Docker Compose script, three containers are started, one for the Neo4j database, one for the NeoDash dashboard and an Nginx server which serves the iframe visualisations available within the dashboard. The database can be accessed by navigating to <http://localhost:7474> in a web browser to access the neo4j browser tool or connecting to ProCogDash via <http://localhost:5005/>. On linux, the compose-run.yml file can be modified (or the run_services.ps1 on Windows) to specify memory allocation for the Neo4j database, which can be adjusted as necessary for your system. Currently, these are not set by the install script, and so will operate with the memory configured in docker. To adjust these parameters add the following lines to the environment section of the compose_run.yaml file (or add as environment parameters in the `run_servcies.ps1` file on Windows):
    - NEO4J_server_memory_heap_initial__size=3600m
    - NEO4J_server_memory_heap_max__size=3600m
    - NEO4J_server_memory_pagecache_size=2g
    - NEO4J_server_jvm_additional=-XX:+ExitOnOutOfMemoryError
6. Access the dashboard. The ProCogDash dashboard is built using NeoDash, a Neo4j plugin. The dashboard can be accessed by connecting to a running instance of the database in Docker at <http://localhost:5005>. The dashboard requires a username and password, which are set to neo4j and procoggraph by default.
7. To stop the database, run the following command:
   Linux/MACOS:
    docker compose -f compose_run.yml down
   Windows:
    Set-ExecutionPolicy Unrestricted
    ./stop_services.ps1
    Set-ExecutionPolicy Restricted

*Neo4j*

Installation instructions for running the database on bare metal, rather than Docker, are described below.

1. Download the latest database flat files from Zenodo <https://zenodo.org/records/13165852/> and clone the ProCogGraph repository:
    git clone m-crown/ProCogGraph
    curl \
   <https://zenodo.org/records/13165852/files/procoggraph_flat_files_v1-0.zip?download=1>
    -o procoggraph_flat_files_v1-0.zip
2. Download and install Neo4j community edition from the <https://neo4j.com/download/>. The database was built using Neo4j version 5.
3. Copy the build script from the repository to the Neo4j database directory (e.g. neo4j-5.4.0) and the database flat files to the import directory:
   cp -r \

/PATH/TO/PROCOGGRAPH_REPOSITORY/nextflow/bin/import_neo4j_data.sh \

/PATH/TO/NEO4J_DATABASE/
cp -r /PATH/TO/DATABASE_FLAT_FILES/* /PATH/TO/NEO4J_DATABASE/import/

1. Run the build script:
   cd /PATH/TO/NEO4J_DATABASE/
   ./import_neo4j_data.sh
2. Start the Neo4j database:
   bin/neo4j start

6. Access the database by navigating to <http://localhost:7474> in a web browser and update the default password (set to user neo4j and password neo4j by default).

7. Access ProCogDash via <http://neodash.graphapp.io/>. The dashboard can be loaded into Neodash by expanding the menu option in the bottom left of the screen, clicking the + icon and importing the dashboard from a JSON file. Upload the file from the repository at procogdash/dashboard.json.

**ProCogGraph Pipeline**

The ProCogGraph pipeline is built using Nextflow for workflow management. To run the pipeline, follow these steps:

- The pipeline utilises data from a number of different sources to build the ProCogGraph database. To begin, prepare a data files directory using the table of data sources available in the ProCogGraph repository.
- Clone this repository and install dependencies:
  git clone m-crown/ProCogGraph
  cd ProCogGraph
  conda env create -f nextflow/envs/environment.yml
- Preprocess RHEA reaction files:
  cd /PATH/TO/DATA_DIR/
  python3 preprocess_rhea.py \

--rhea_ec_mapping rhea2ec.tsv \

--rhea_reaction_directions rhea-directions.tsv \

--rd_dir rd/ \

--outdir . \

--chebi_names chebi_names.tsv.gz

- Produce final manifest file of structures to be processed:
  python3 download_mmcif.py \

--sifts_file /PATH/TO/DATA_DIR/pdb_chain_enzyme.tsv.gz \

--assemblies_file /PATH/TO/DATA_DIR/assemblies_data.csv.gz

--chunk_size 100 --output_dir /PATH/TO/STRUCTURES_DIR

- Run the nextflow pipeline. To configure the nextflow pipeline, the nextflow.config file within the repository should be modified. A SLURM cluster profile, specific for the development of the pipeline within the Bashton Group at Northumbria, is included called 'crick'. The standard profile is designed for running the pipeline on a local machine, and is by default configured with a large amount of memory and CPU resources. This should be adjusted before running. Four additional parameters must be set specific to the user's environment:
   - params.data_dir - the path to the data directory created including data files described above.
   - params.cache_in - the path to the cache directory for the pipeline, if pipeline has been run previously.
   - params.output_dir - the desired output directory.
   - params.manifest - the path to the manifest file created in step 3.

  cd /PATH/TO/PROCOGGRAPH_REPOSITORY/nextflow
  nextflow run main.nf -resume -profile standard

### S2. ProCogDash Implementation.

The underlying ProCogGraph database can be explored using ProCogDash - a NeoDash dashboard which collects various sets of useful Cypher queries into a single source, running either locally or accessible from <http://neodash.graphapp.io/>. In contrast to past web versions of PROCOGNATE, similarity score cutoffs are user-customisable, from the minimum cutoff of 0.4 PARITY score up to 1.0/perfect similarity. When exploring the range of ligands above the minimum cutoff, a user may want to search with a higher cutoff if multiple potential cognate ligands are identified for a PDB ligand, for example, to ensure docking studies utilise the most similar cognate ligand structure.

The dashboard contains five key visualisation modes as UI tabs: PDB, Cognate Ligand, PDB Ligand, Domain and EC. The homepage provides summary statistics for the number of structures and ligands represented in the current version of the graph and the number of cognate ligand matches for the currently specified cutoff. From the search page, global search settings can be specified, which consists of a similarity score cutoff for cognate ligand matching, and the domain database type to be searched against. A filter can also be applied to all cognate ligand matches to specify which cognate ligand matches should be considered:

- “All”, in which all bound entities for a PDB are displayed in the interaction table, regardless of whether they have a mapping to a cognate ligand.
- “Any”, in which all cognate ligand matches for a bound entity which are above the scoring threshold are presented.
- “Best” in which only cognate ligands with the highest score for a given bound entity are shown.

It should be noted that even when set to “Best”, a bound entity may have matches to multiple cognate ligands with the same maximum score. ProCogGraph is designed to serve as an information source, and so does not make an effort to select a particular best match as the “Best” best match, instead leaving this up to the user.

Next, users can initiate a search for one of each of the five visualisation modes. The PDB search box is used to search for a structure, and a PDB ID can be matched from any partial searches via a dropdown list. A clickable link is then presented next to the PDB search box, which takes you to the PDB visualisation mode.

On the PDB exploration page, a summary report is generated and presented, alongside a table of interactions between domains and bound entities in the structure, and the mapping of bound entities to cognate ligands.

Alongside the domain interaction table, an embedded iframe allows visualisation of the interacting residues between bound entities and domain residues in the structure, using PDBe-Molstar. In this visualisation, a list of interacting residues belonging to the selected domain are presented alongside the structure, with residues from the currently focussed domain highlighted in blue, and other domain residues highlighted in purple - this view is shown in Supplementary Figure 10.

The PARITY score between cognate ligands and bound entities can also be viewed, showing both the MCS match and the atom matches, making up the PARITY score - visualised with RDKit JS. An alternative to using RDKit JS for visualisation is the generation of image files for each structure. However, to view MCS structures and atom matches for all combinations of PDB and cognate ligands would necessitate a large amount of disk storage and significant computation. By dynamically generating these visualisations, the overall footprint of ProCogDash is minimised. The iframe visualisations are loaded from an Nginx server running in the distributed Docker image if running locally. Visualisation of the full contact annotation from PDBe-Arpeggio and SIFTS domain annotations is not possible with Molstar viewer, and so alongside this, ProCogGraph contains a script to generate Pymol sessions for each structure, displaying contacts and domain information on the assemblies using the information produced during pipeline execution.

For each domain listed in the PDB structure page, breakout links are accessible to a domain summary page (domains can also be searched for directly from the search page using the domain search box). This page includes a summary report of the number of ligands the domain is known to interact with, together with links to the external domain annotation. The report also summarises interactions for a domain at a “group” level, which varies depending on the domain database being examined; for example, in the CATH/Gene3D/SCOP/SUPERFAMILY, the group level is Superfamily, and for Pfam, it is the family level. Summaries are presented on the following:

- Group interactions table: lists all cognate ligands a group level are known to interact with in the database, together with the number of domains that interact with the ligand.
- Domain Contexts: This query lists the contexts in which a domain interacts with a ligand i.e, the other domains involved in the interaction and their interaction modes.
- Domain Cognate Ligand breakdowns: table lists the cognate ligands the specific domain searched for interacts with, and the percentage of the overall group the domain belongs to which also interact with the ligand. This is useful for identifying if the cognate ligand a domain binds to reflects typical superfamily activity or is an outlier.

Cognate and PDB ligands can be viewed in detail through breakout from the PDB structure page view. Both pages contain a similar set of results tables, including an RDKit.js visualisation of the ligand structure, a summary report detailing the cognate ligand database cross-references or the number of times a PDB ligand has been observed in the database, and the domain interactions that are observed for a ligand. Additionally, PDB ligands contain link tables to cognate ligand mappings. As with domains, PDB and cognate ligands can also be searched for directly from the search page, either by hetcode or name for PDB ligands, or database ID (format DB:ID) or name for cognate ligand.

When searching for an EC number, results are aggregated and links presented to relevant structures, cognate and PDB ligands, together with a summary of domains known to interact with ligands In this reaction. In addition the reactions associated with the EC number within the RHEA database are visualised using RDKit-js, allowing for dynamic generation based on the reaction smiles strings associated with the EC nodes in the graph.

The interlinked nature of the various results pages and their associated query results enable an intuitive and easy-to-use tool to drive analyses using ProCogGraph. An important aspect of any tool is the user-community, and one of the primary reasons for using Neo4J as a DBMS was the Neodash plugin which has been used to create ProCogDash. This plugin enables easy extensibility by users to create their own visualisations. Using a local version of the graph, which can be easily set up using the instructions provided in the repository, a user can connect to this graph in a few clicks and create a fresh dashboard that can be populated with their own research queries.

Every result presented in ProCogDash is generated using a Cypher query, which are contained within the neodash dashboard, and which is stored as a node within the database, allowing it to be versioned and distributed alongside the database itself. In addition to this, all queries are also made available within the repository as a single YAML file, where each query contains additional comments describing the underlying process.

Overall, the ProCogDash dashboard enables rapid and rich interrogation of the underlying database, in a highly adaptable and customisable manner due to the flexible Cypher queries underpinning the dashboard, which allow for high levels of filtering and customisation of the returned data depending on a user’s use case.

### S3. Common ligands with no cognate mapping

The most frequently observed bound entity descriptor with no cognate ligand match is Chlorophyll A (present in structures with EC reactions 1.97.1.12, 7.1.1.6 and 1.10.3.9). In these reactions, Chlorophyll A is referenced in the EC reaction comments text but is not present as a component of the reaction itself. Without inclusion in the reaction, ProCogGraph cannot assign this ligand as cognate, so remains unannotated. PC1, 3PE, PLX and PEK are all phospholipids, frequently found in liposomes and membranes, but not part of the enzyme reaction, hence they remain unmapped. Lipidic cubic phase crystallisation is the most commonly used method for crystallising membrane proteins, and varying mixes of all of these phospholipids have been studied for their effects on the structure of membranes formed in this crystallisation method [(Caffrey 2015)](https://paperpile.com/c/134AzV/H07c). Additionally, tetrafluoroaluminate and beryllium trifluoride ions are often used in combination with ADP/GDP to simulate binding of ATP/GTP binding sites in proteins [(Bigay *et al.* 1987)](https://paperpile.com/c/134AzV/E0gh), which act as an analogue of the nucleotide triphosphate gamma phosphate [(Pellegrini *et al.* 2024)](https://paperpile.com/c/134AzV/feKN). In many cases, the metal fluoride is left unmatched whilst there is a cognate ligand match between ADP and cognate ATP *e*.*g*. in PDBs 1MMD, 4TYN and 3GLG.

### S4. EC Coverage and Cognate Ligand Database Overlap

ProCogGraph includes nodes for all EC numbers in ENZYME (at the time of development 6,753 EC numbers) regardless of whether they have a cognate ligand or protein chain annotation in the database. This allows for simple expansion in future versions and can be used to identify areas of under-annotation. A total of 556/6,753 (8.2%) EC numbers have no cognate ligand mapping within the database. The majority of these (483/556, 86.9%) belong to EC subclass group 3.1, 3.2 or 3.4. The most incomplete EC class in ProCogGraph is EC 3.4, Hydrolases Acting on peptide bonds (Peptidases), reflecting the fact that in ProCogGraph, polypeptide ligands are not considered.

ProCogGraph aggregates structurally identical cognate ligands (after preprocessing) into single entries, to reduce duplication and minimise computational overhead during similarity searching. A total of 8,589 unique ligands are present in the database, each of which may have one or more cross references to a cognate ligand database (mean number of database cross references per ligand = 2.25). The best represented cognate ligand database in the dataset is ChEBI, for which IDs are available for 7,087 cognate ligands, followed by KEGG with 6,450 cognate ligands. Considering pairwise occurrences of different database cross-references, more than 50% of cognate ligands contain cross references to two cognate ligand databases:

- ChEBI + KEGG: 5,509 cognate ligands
- ChEBI + Pubchem: 4,742 cognate ligands
- KEGG + Pubchem: 5,031 cognate ligands

However, when considering combinations of three different domain database cross references for a ligand, less than 100 cognate ligands have a combination of three different cognate ligand databases, except the combination of ChEBI, KEGG and Pubchem where we observe 4,703 cognate ligands. Improving the cognate ligand cross referencing in future versions of ProCogGraph and integrating more cognate ligand databases, together with manual annotation of “ProCogGraph” ligands will help to improve the overall annotation of domain-ligand interactions with ProCogGraph, feeding this back to source databases will require collaboration with relevant database maintainers.
