# Supplementary material for: ProCogGraph: a graph-based mapping of cognate ligand domain interactions
Source: Bioinform Adv. 2024 Oct 22;4(1):vbae161. doi: 10.1093/bioadv/vbae161 (PMC11561043; doi:10.1093/bioadv/vbae161)
Supplement: vbae161_Supplementary_Data [file vbae161_supplementary_data.zip › RESUB_ProCogGraph Paper Supplementary Tables.docx]

**Supplementary Table 1: Domain interaction modes in ProCogGraph**. One or more domains may interact with a ligand, and depending on the percentage of contacts a domain provides to the interaction, a different interaction mode is assigned. Note one domain may interact with multiple ligands with different interaction modes.

| **# Interacting Domains** | **Domain % Contacts** | **Interaction Mode** | **Description** | **% Domains Exhibiting Interaction Mode** | | | | | |
| --- | --- | --- | --- | --- | --- | --- | --- | --- | --- |
|  |  |  |  | **CATH** | **SCOP** | **Pfam** | **SUPERFAMILY** | **G3DSA** | **SCOP2** |
| 1 | 100% | Exclusive | A single domain contacts the ligand. | 77.23 | 84.03 | 86.24 | 89.56 | 84.17 | 88.95 |
| 2+ | ≥90% | Dominant | Two or more domains interact, and this domain dominates in terms of contact % | 1.50 | 1.02 | 0.63 | 0.72 | 0.51 | 0.57 |
| 2+ | <10% | Minor | Two or more domains interact, and this domain only plays a minor role in the interaction interface | 1.86 | 1.22 | 0.94 | 0.92 | 1.35 | 0.79 |
| 2+ | 10-90% | Major | Two or more domains interact, and this domain is the only domain with more than 10% contacts (i.e. other domains are all minor) | 0.03 | 0.002 | 0.03 | 0.02 | 0.01 | 0.03 |
| 2+ | 50-90% | Major Partner | Two or more non-minor domains interact, and this domain provides more than 50% of contacts. | 21.21 | 16.79 | 16.02 | 15.28 | 18.35 | 15.43 |
| 2+ | 10-50% | Partner | Two or more non-minor domains interact, and this domain provides up to 50% of contacts. | 22.90 | 17.72 | 15.85 | 15.27 | 16.77 | 15.98 |

**Supplementary Table 2: Comparison of PROCOGNATE v1.6 (PCN) and ProCogGraph v1.0 (PCG) coverage for various domain databases**. For CATH (v4.2.0) and Gene3D (via InterPro v96.0) databases group level is Homologous Superfamily, for SCOP (v1.75), SCOP2 (v2022-06-29) and Superfamily (via InterPro v96.0), the group level is Superfamily and for Pfam (via InterPro 96.0) the group level is Family. Note: SCOP (v1.75) was last updated in June 2009, resulting in lower coverage.

|  | **CATH** | | **SCOP 1.75** | | **Pfam** | | **SUPERFAMILY** | | **Gene3D** | | **SCOP2** | |
| --- | --- | --- | --- | --- | --- | --- | --- | --- | --- | --- | --- | --- |
|  | **PCN** | **PCG** | **PCN** | **PCG** | **PCN** | **PCG** | **PCN** | **PCG** | **PCN** | **PCG** | **PCN** | **PCG** |
| **PDB Entries In PCN/PCG** | 5,553 | 61,473 | 6,275 | 17,166 | 7,895 | 89,805 | n/a | 74,473 | n/a | 60,500 | n/a | 64,945 |
| **Num Groups** | 427 | 1,907 | 347 | 762 | 7,80 | 3,960 | n/a | 874 | n/a | 1,148 | n/a | 1,054 |
| **EC numbers (only absolutely defined)** | 750 | 2,831 | 873 | 1,694 | 996 | 3,282 | n/a | 2,775 | n/a | 2,435 | n/a | 2,292 |
| **EC Numbers (including expanded partial)** | n/a | 6,652 | n/a | 6,313 | n/a | 6,689 | n/a | 6,651 | n/a | 6,648 | n/a | 6,606 |
| **Ligands In Database** | n/a | 297,547 | n/a | 76,446 | n/a | 433,442 | n/a | 359,828 | n/a | 293,741 | n/a | 309,522 |
| **Ligands with Mapping (0.4 cutoff)** | 24,337 | 104,989 | 26,948 | 28,823 | 32,450 | 151,529 | n/a | 125,909 | n/a | 102,807 | n/a | 101,297 |
| **Number of cognate ligands mapped** | n/a | 2296 | n/a | 1,189 | n/a | 2710 | n/a | 2,340 | n/a | 2,098 | n/a | 2,114 |

**Supplementary Table 3: Top 10 PDB Ligands with no cognate ligand mapping in ProCogGraph**

| **Het Code** | **Name** | **# Bound Entities** | **Structure** |
| --- | --- | --- | --- |
| CLA | CHLOROPHYLL A | 1,102 | 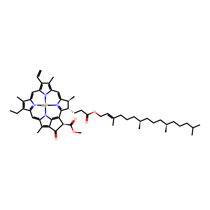 |
| CD | CADMIUM ION | 642 | 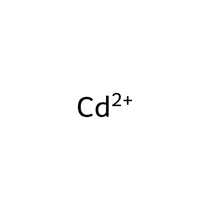 |
| PC1 | 1,2-DIACYL-SN-GLYCERO-3-PHOSPHOCHOLINE | 537 | 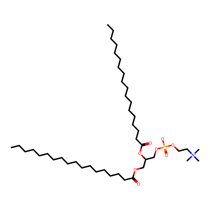 |
| 3PE | 1,2-DIACYL-SN-GLYCERO-3-PHOSPHOETHANOLAMINE | 428 | 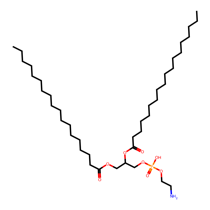 |
| BEF | BERYLLIUM TRIFLUORIDE ION | 409 | 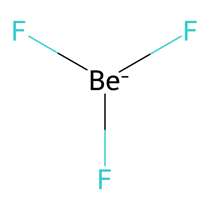 |
| PLX | (9R,11S)-9-({[(1S)-1-HYDROXYHEXADECYL]OXY}METHYL)-2,2-DIMETHYL-5,7,10-TRIOXA-2LAMBDA~5~-AZA-6LAMBDA~5~-PHOSPHAOCTACOSANE-6,6,11-TRIOL | 366 | 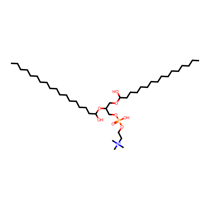 |
| PEK | (1S)-2-{[(2-AMINOETHOXY)(HYDROXY)PHOSPHORYL]OXY}-1-[(STEAROYLOXY)METHYL]ETHYL (5E,8E,11E,14E)-ICOSA-5,8,11,14-TETRAENOATE | 262 | 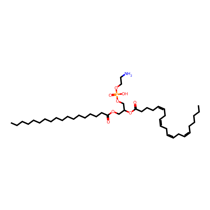 |
| TGL | TRISTEAROYLGLYCEROL | 260 | 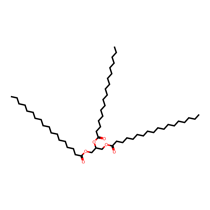 |
| ALF | TETRAFLUOROALUMINATE ION | 245 | 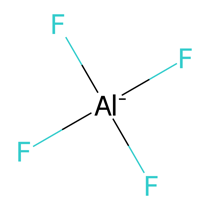 |
| CUA | DINUCLEAR COPPER ION | 227 | 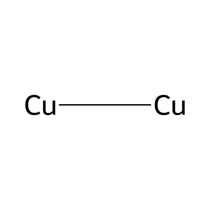 |

**Supplementary Table 4: The ten most specialised and ten most generalised superfamilies, according to their mean intra-group similarity**. The number of unique EC numbers which domains in these superfamilies are annotated with is shown in the final column. Generalised ligands have a higher number of EC numbers associated

| **Specialised/ Generalised** | **CATH Homologous Superfamily** | **# Cognate Ligands** | **Mean Similarity** | **Similarity St. Dev.** | **# Unique EC Numbers** |
| --- | --- | --- | --- | --- | --- |
| Specialised | Single alpha-helices involved in coiled-coils or other helix-helix interfaces | 4 | 1.00 | 0.00 | 1 |
|  | GI Alpha 1, domain 2-like | 4 | 0.97 | 0.02 | 5 |
|  | Fatty acid synthase; domain 2 | 4 | 0.97 | 0.02 | 8 |
|  | Lyase 2-enoyl-coa Hydratase, Chain A, domain 2 | 8 | 0.96 | 0.02 | 5 |
|  | Nitrogenase Molybdenum-iron Protein, subunit B; domain 4 | 5 | 0.95 | 0.04 | 1 |
|  | Probable inorganic polyphosphate/atp-NAD kinase; domain 2 | 5 | 0.94 | 0.03 | 2 |
|  | oxygen-dependent coproporphyrinogen oxidase | 12 | 0.91 | 0.07 | 4 |
|  | Imidazole glycerol phosphate dehydratase; domain 1 | 4 | 0.88 | 0.07 | 1 |
|  | Alpha-Beta Plaits | 7 | 0.87 | 0.06 | 10 |
|  | F420-dependent methylenetetrahydromethanopterin dehydrogenase (MTD) | 6 | 0.86 | 0.08 | 1 |
| Generalised | main proteinase (3clpro) structure, domain 3 | 4 | 0.11 | 0.08 | 468 |
|  | Ribonucleotide Reductase, subunit A | 24 | 0.14 | 0.15 | 1387 |
|  | Cytochrome c oxidase-like, subunit I domain | 7 | 0.14 | 0.23 | 5 |
|  | Ferritin | 20 | 0.14 | 0.18 | 268 |
|  | Copper amine oxidase, catalytic domain | 18 | 0.15 | 0.18 | 24 |
|  | Cytochrome c-like domain | 21 | 0.16 | 0.18 | 1061 |
|  | Sulfite Reductase Hemoprotein, domain 1 | 7 | 0.16 | 0.23 | 4 |
|  | Nitrile hydratase, beta subunit | 5 | 0.16 | 0.19 | 1 |
|  | Nitrile hydratase alpha /Thiocyanate hydrolase gamma | 5 | 0.16 | 0.19 | 1 |
|  | Cupredoxins - blue copper proteins | 19 | 0.17 | 0.19 | 27 |

**Supplementary Table 5: Top 20 most promiscuous domains as defined by number of different cognate ligands a superfamily interacts with**.

| **CATH Homologous Superfamily** | **# Cognate Ligands** | **Mean Similarity** | **Similarity St. Dev.** |
| --- | --- | --- | --- |
| NAD(P)-binding Rossmann-like Domain | 338 | 0.29 | 0.19 |
| Aldolase class I | 186 | 0.31 | 0.20 |
| NADP-dependent oxidoreductase domain | 157 | 0.30 | 0.19 |
| alpha/beta hydrolase | 153 | 0.25 | 0.16 |
| Cytochrome P450 | 139 | 0.27 | 0.19 |
| FAD/NAD(P)-binding domain | 128 | 0.26 | 0.18 |
| P-loop containing nucleotide triphosphate hydrolases | 116 | 0.33 | 0.25 |
| Vaccinia Virus protein VP39 | 108 | 0.28 | 0.18 |
| Type I PLP-dependent aspartate aminotransferase-like (Major domain) | 100 | 0.35 | 0.17 |
| Glycosidases | 91 | 0.48 | 0.23 |
| Metal-dependent hydrolases | 84 | 0.28 | 0.19 |
| Aspartate Aminotransferase, domain 1 | 72 | 0.34 | 0.18 |
| Medium-chain alcohol dehydrogenases, catalytic domain | 69 | 0.24 | 0.17 |
| Dihydrodipicolinate Reductase; domain 2 | 68 | 0.35 | 0.19 |
| Spore Coat Polysaccharide Biosynthesis Protein SpsA; Chain A | 65 | 0.55 | 0.22 |
| Nucleoside Triphosphate Pyrophosphohydrolase | 57 | 0.51 | 0.34 |
| HUPs | 56 | 0.38 | 0.25 |
| Ribonuclease H-like superfamily/Ribonuclease H | 56 | 0.25 | 0.15 |
| Aminopeptidase | 53 | 0.25 | 0.19 |
| Alpha-Beta Plaits | 51 | 0.30 | 0.20 |
