# Supplementary material for: ProCogGraph: a graph-based mapping of cognate ligand domain interactions
Source: Bioinform Adv. 2024 Oct 22;4(1):vbae161. doi: 10.1093/bioadv/vbae161 (PMC11561043; doi:10.1093/bioadv/vbae161)
Supplement: vbae161_Supplementary_Data [file vbae161_supplementary_data.zip › RESUB_ProCogGraph Paper Supplementary Figures.docx]

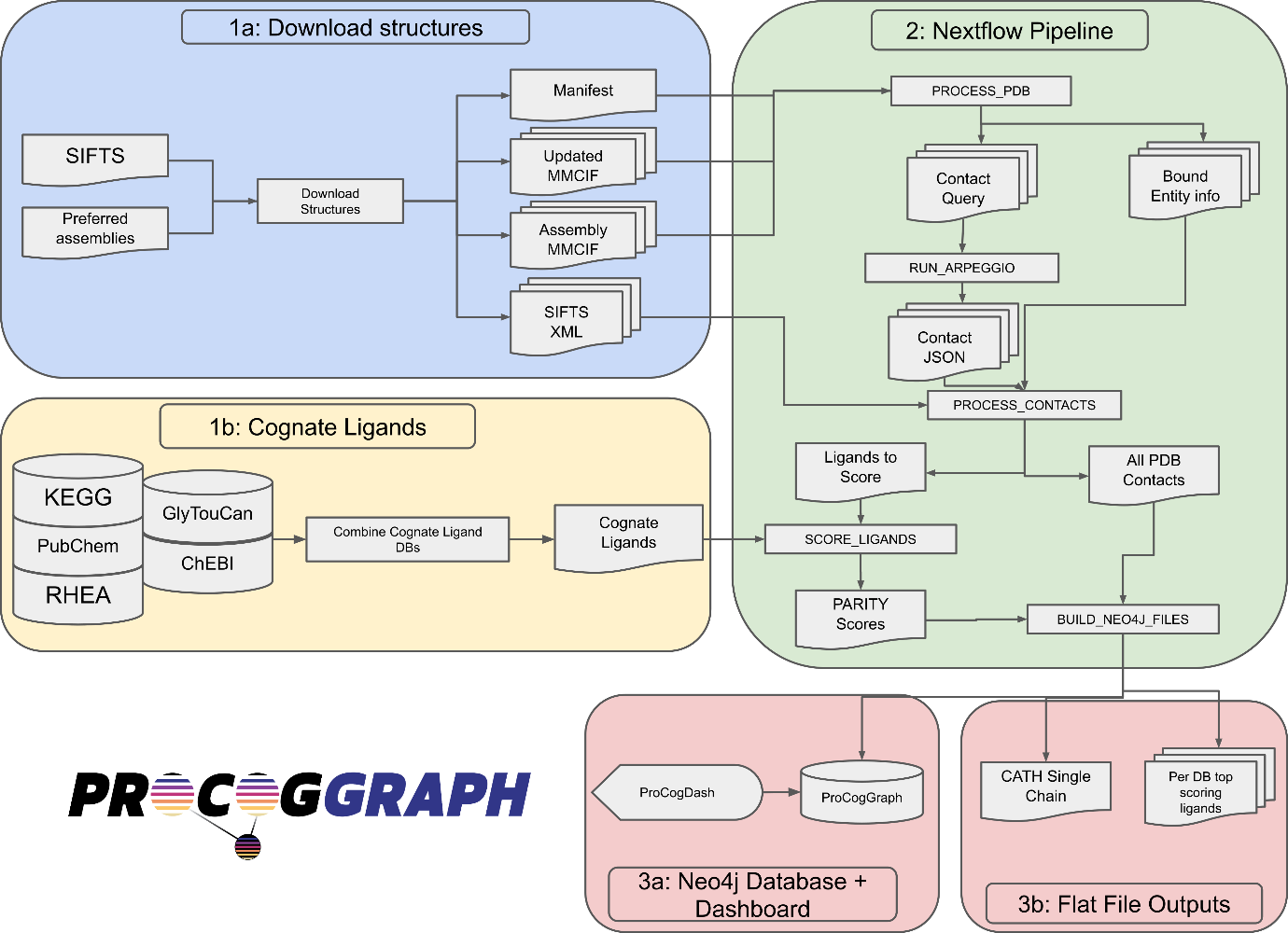


**Supplementary Figure 1: The ProCogGraph Pipeline**. 1a) Updated and protonated MMCIF files and SIFTS XML annotations are downloaded from EBI servers (PDBe and modelserver). 2) The Nextflow pipeline is executed, processing each PDB structure to determine bound ligands in the assembly and generate query files for Arpeggio. The generated ligand contacts are then parsed into domain-specific ranges, and interaction modes between domains and ligands are determined. 3) Finally, Neo4j format files and flat file outputs are generated for the ProCogGraph database.


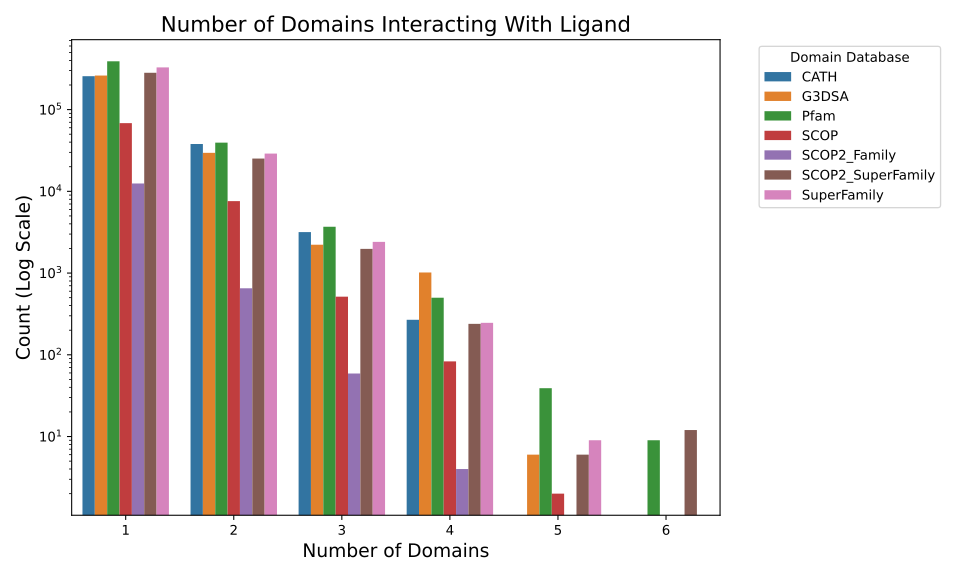


**Supplementary Figure 2: Breakdown of number of domains interacting with bound entities in ProCogGraph, for each domain database**. Most interactions to a bound entity involve one or two domains, with 3+ multi-domain interactions less frequent. Five and six domain interactions are observed in a few, but not all domain databases.


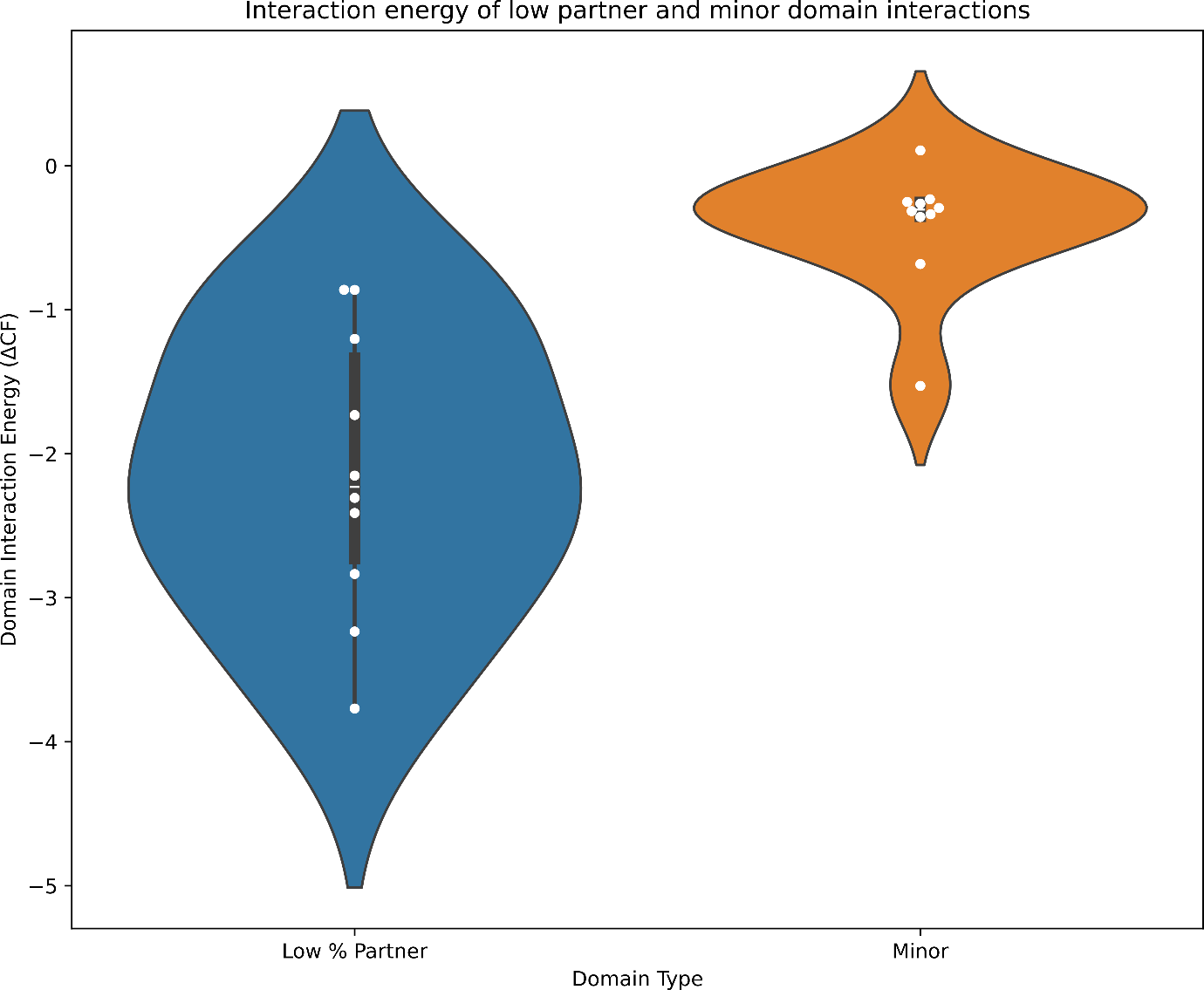


**Supplementary Figure 3: Mean interaction favourability of minor and low % partner contact domains to their bound ligands**. Low % partner domains show significantly (Independent t-test, t=-5.05, p < 0.001) stronger interaction to ligands than minor domains. Domain interaction energy is measured as the sum of energies calculated with Surfaces’ complementarity function (CF), and is a pseudo-energetic measurement (no units).


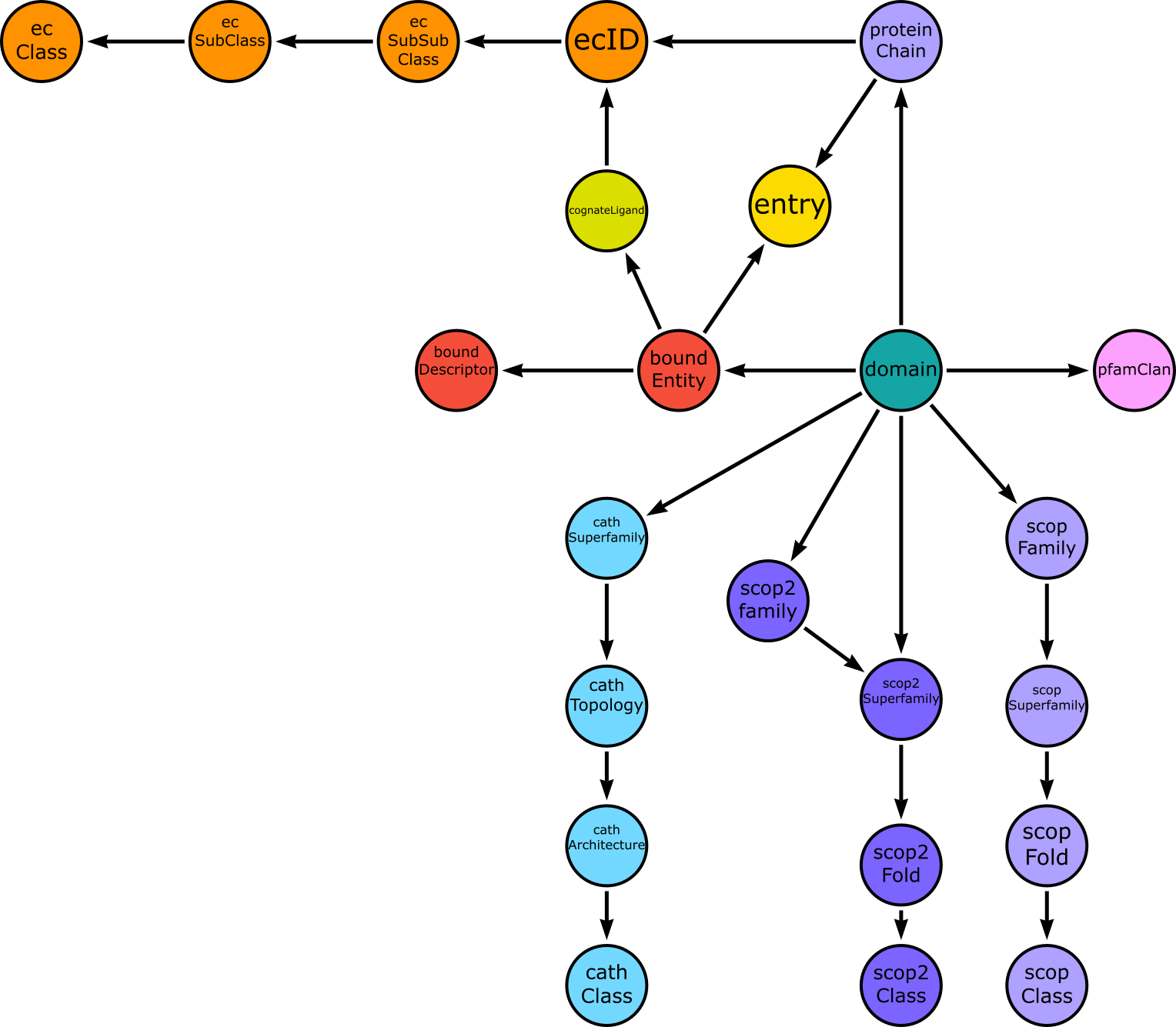


**Supplementary Figure 4: The ProCogGraph schema**. The schema can be split at the highest level into four main node types: Enzyme Class, Cognate Ligand, Bound Entity and Domain. Further granularity is then available for the relationships of these nodes, which annotates hierarchical relationships in, for example, the Enzyme Classification system, and also the interrelationship between domains, enzyme classification and cognate ligand enzyme classification.


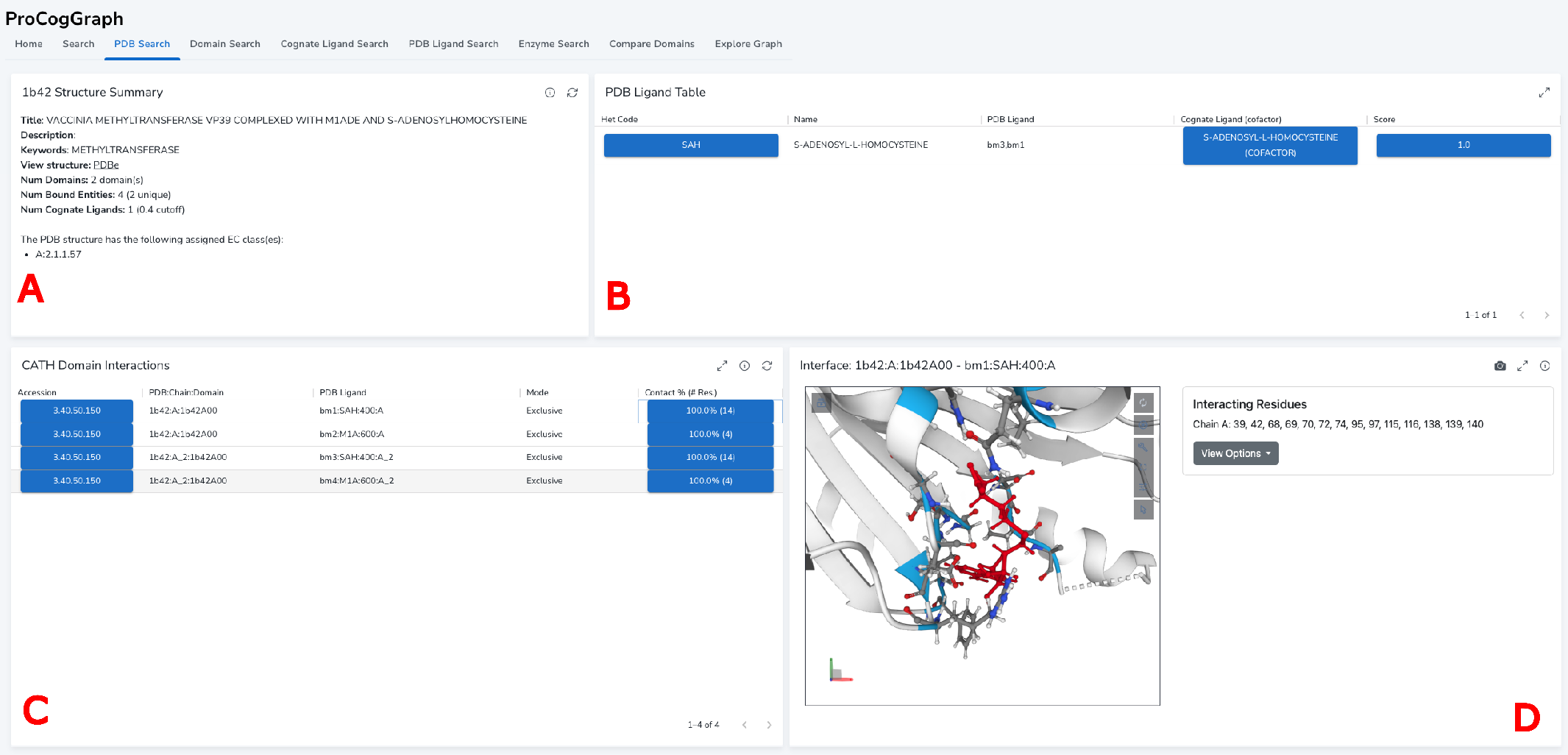


**Supplementary Figure 5: View of the ProCogGraph dashboard, PDB view**. The PDB structure 1B42 has been selected from the search page, resulting in a summary reported being presented. A: Summary text is derived from the mmCIF file including title, description, and keywords if available. The number of domains present from the currently selected database (here CATH) is stated together with the number of unique bound entities in the structure. The current PARITY score cutoff is noted together with the number of cognate ligand matches found. B: The unique bound entities in the structure are listed, including Het Code, name and specific occurrences e.g. bound molecule 1 and 2. Clicking the het code for a ligand navigates and populates a summary report for the bound entity including structures it occurs in and a visualisation of its structure. The cognate ligand matches if available for a ligand are listed (click to view Cognate Ligand summary page) and the similarity score. Clicking the similarity score displays the two structures together with their MCS in the iFrame section of panel D. C: The domain interactions table lists each domain, the ligand it interacts with and the mode of interaction. Clicking on the domain sends users to a domain summary page, and clicking on the contact % column for a domain loads an embedded Molstar viewer display of the domain-ligand interaction. D: iframe display section – when clicking on a cognate ligand score, the viewer displays the structure of the compared ligands and the score between them together with the MCS highlighted. When clicking on a domain interaction, a Molstar viewer display of the interaction is shown, with interacting residues from the focussed domain highlighted in blue, all other interactions highlighted in purple.


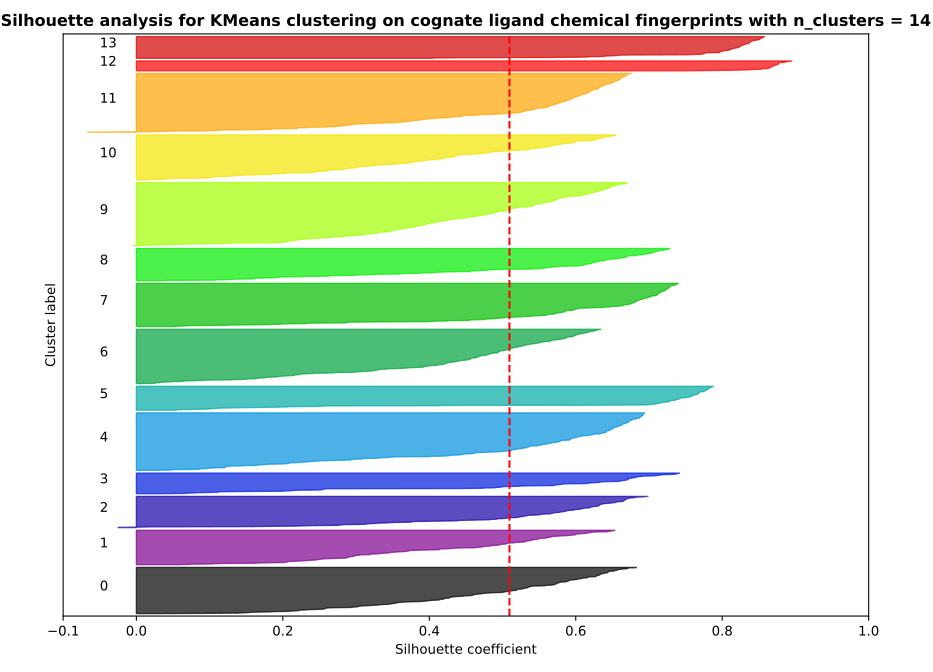


**Supplementary Figure 6: Silhouette analysis of KMeans Clustering on Chemical Similarity of Cognate Ligands, showing the silhouette coefficient for each cluster with n=14 clusters. Red dashed line represents the average silhouette score (0.51) across all clusters**. Silhouette analysis is used to determine the appropriate cluster number for cognate ligand clustering based on chemical fingerprints. The Silhouette analysis shown is for the cluster number which provided the highest average silhouette score. Each bar represents a ligand, and its associated silhouette score. It can be seen from the figure that most ligands have a high positive silhouette score, which indicates that the ligand is well matched to its cluster and does not cluster well with other clusters. The tails associated with each cluster’s bar plots indicate that their separation is not high condensed, which is to be expected as some ligands, representing the substrate space, may have features that make them suited to belonging to multiple clusters.


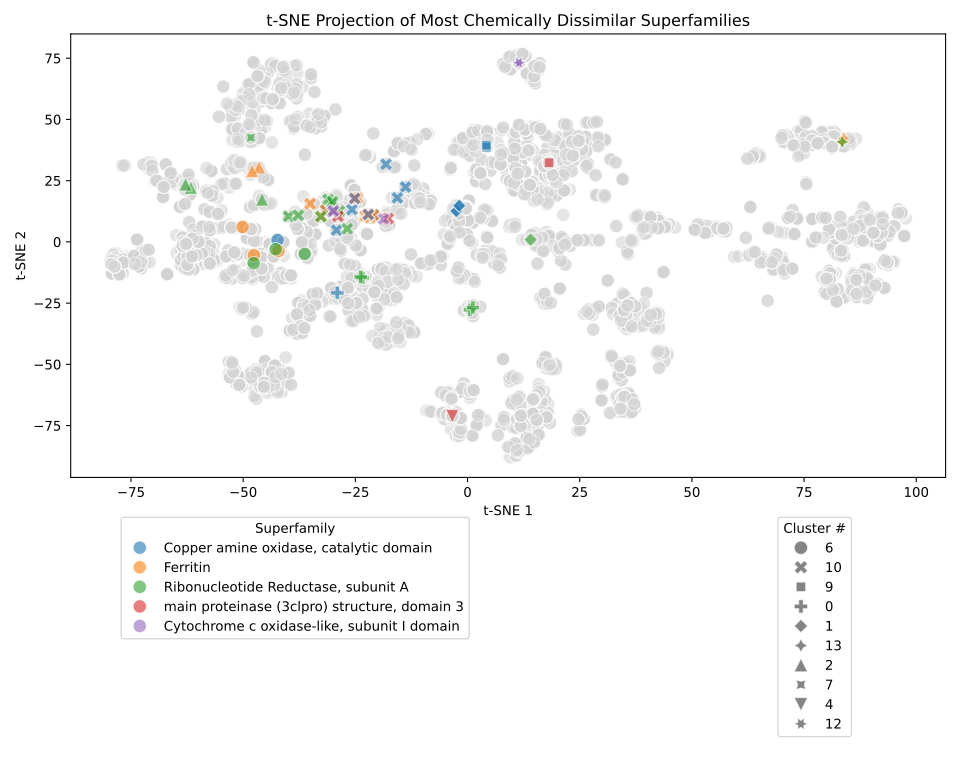


**Supplementary Figure 7: t-SNE visualisation of the five most “generalised” superfamilies with the highest intra-superfamily chemical dissimilarity**. Cognate ligands are coloured according to the superfamily they belong to against a backdrop of all unique cognate ligands in the mapping. Ligands from generalised superfamilies are assigned distinct shapes according to the cluster they were assigned to.


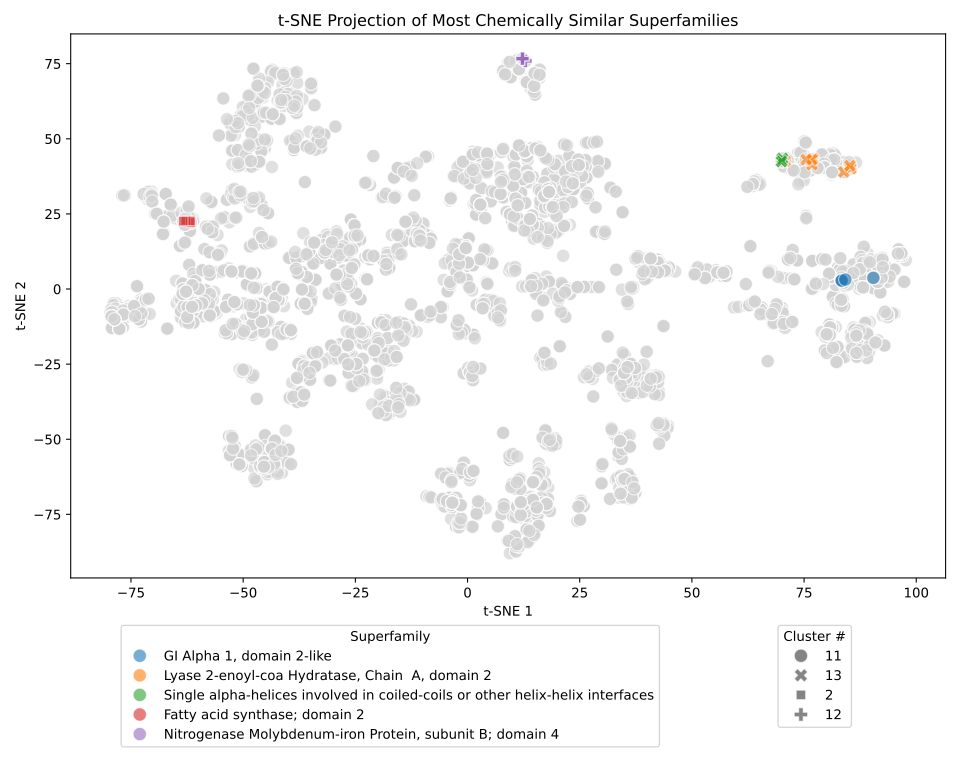


**Supplementary Figure 8: t-SNE visualisation of the five most “specialised” superfamilies with the highest intra-superfamily chemical similarity**. Cognate ligands are coloured according to the superfamily they belong to against a backdrop of all unique cognate ligands in the mapping. Ligands from specialised superfamilies are assigned distinct shapes according to the cluster they were assigned to.


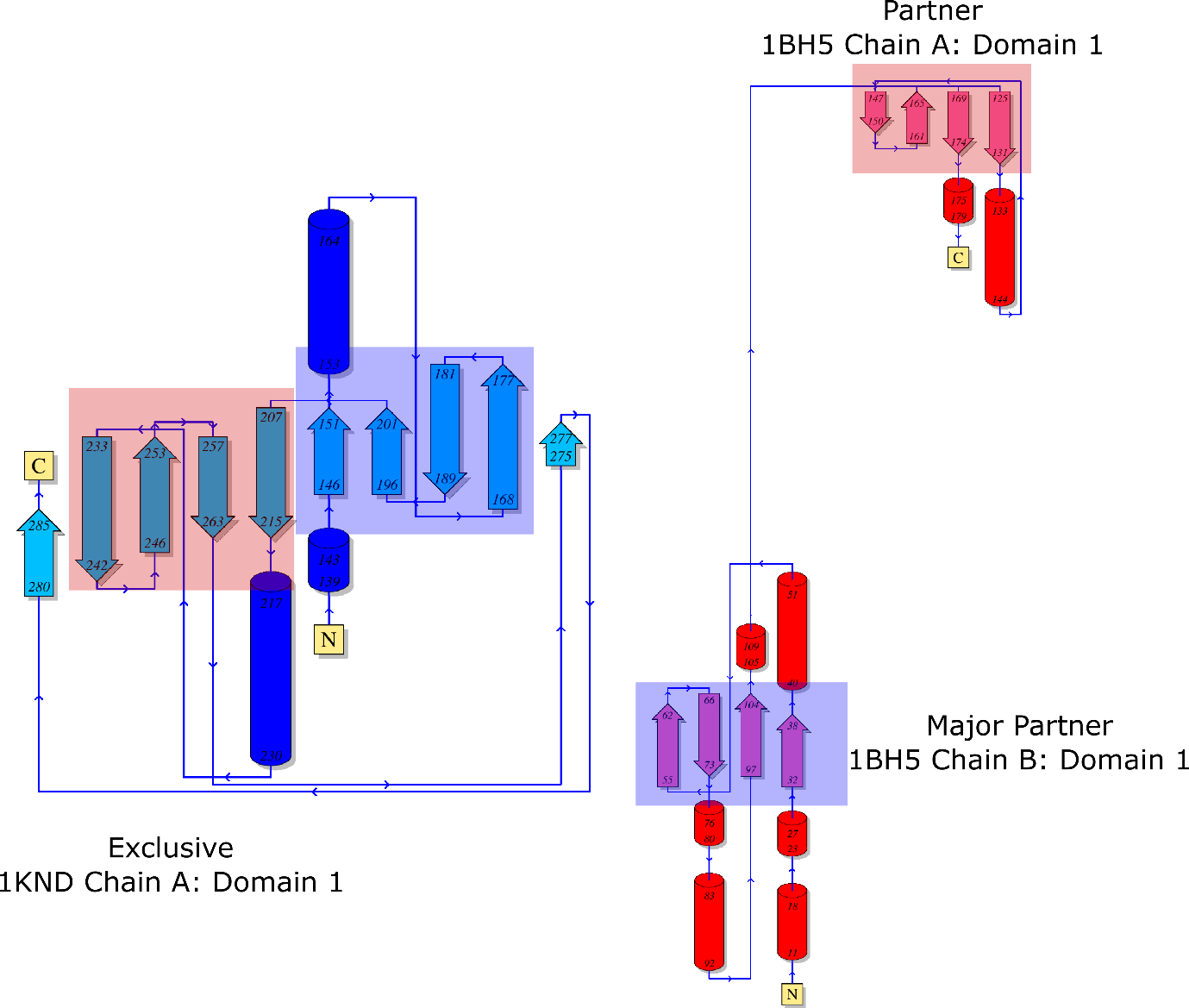


**Supplementary Figure 9: Domain organisation in exclusive and partner binding domains of superfamily 3.10.180.10**. Left: Domain organisation in structure 1KND domain 1, which binds its ligand exclusively. Right: Domain organisation in structure 1BH5 domain 1, which binds its ligand in a partner interaction between two instances of the domain, in chain A and chain B. Blue highlighted beta strands in 1KND domain correspond to beta strands involved in interaction from chain B in 1BH5. Beta strands highlighted in red in 1KND correspond to beta strands involved in interaction from chain A in 1BH5. Figure composited from images taken from PDBsum.


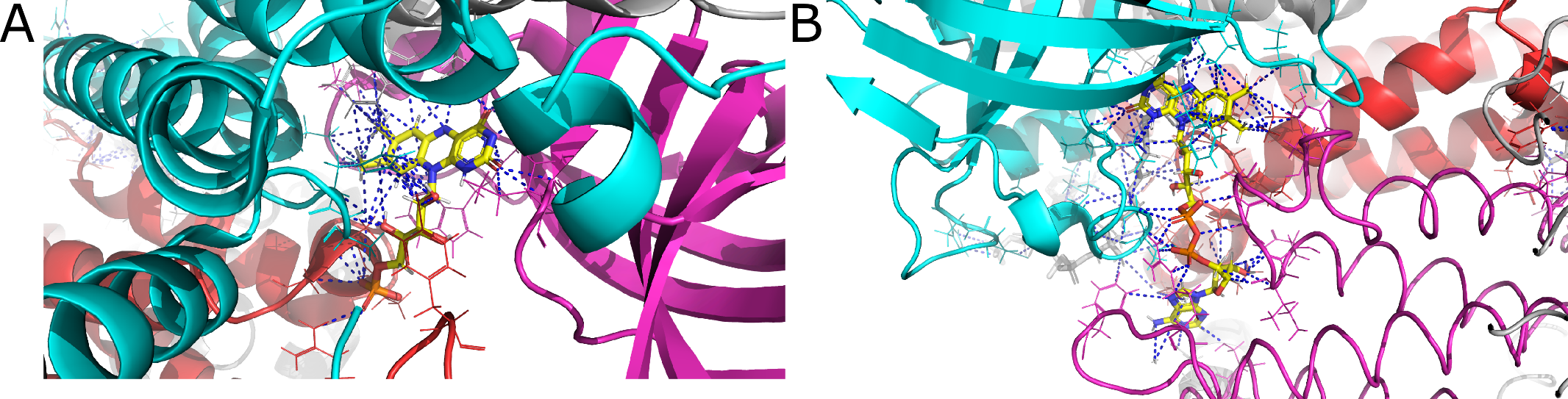


**Supplementary Figure 10: FAD/FMN binding pocket formed by three domains**. Interatomic contacts identified between residues are shown with blue dashed lines, residues involved in interaction are shown as wireframe molecules and bound ligands are shown in stick representations with carbons coloured yellow. A: FMN binding pocket within a p-hydroxyphenylacetate hydroxylase (PDB 2JBS). Red: Butyryl-CoA Dehydrogenase, subunit A, domain 3 (CATH 1.20.140.10) domain, chain A. Magenta: Domain Butyryl-CoA Dehydrogenase, subunit A, domain 2 (CATH 2.40.110.10), chain D. Cyan: Butyryl-CoA Dehydrogenase, subunit A, domain 3 (CATH 1.20.140.10) domain. Ligand Flavin Mononucleotide matches to cognate ligand FAD, similarity = 0.59. B: FAD binding pocket within a butyryl-CoA dehydrogenase (PDB 1BUC). Magenta: Domain Butyryl-CoA Dehydrogenase, subunit A, domain 3 (CATH 1.20.140.10), Cyan: Domain Butyryl-CoA Dehydrogenase, subunit A, domain 2 (CATH 2.40.110.10), Red: Domain Butyryl-CoA Dehydrogenase, subunit A, domain 3 (CATH 1.20.140.10). Ligand FAD matches to cognate ligand FAD with perfect similarity
